# Palmitoylation in cancer: decoding its roles in signal transduction, tumor immunity, and emerging therapeutic opportunities

**DOI:** 10.3389/fimmu.2025.1640016

**Published:** 2025-09-04

**Authors:** Qiguang Lu, Jiasheng Wu, Xiaoyan Yu, Juanjuan Qian, Zhengwei Song

**Affiliations:** ^1^ Department of Respiratory Medicine, The Second Affiliated Hospital of Jiaxing University, Jiaxing, Zhejiang, China; ^2^ Department of Surgery, The Second Affiliated Hospital of Jiaxing University, Jiaxing, Zhejiang, China

**Keywords:** palmitoylation, tumorigenesis, signal transduction, cancer therapy, post-translational modification

## Abstract

Palmitoylation, a reversible post-translational modification involving the attachment of palmitic acid to cysteine residues of proteins, plays a critical role in the regulation of protein localization, stability, and function. Recent studies have revealed its significant involvement in various oncogenic processes, including tumor initiation, progression, metastasis, and immune evasion. This review comprehensively explores the molecular mechanisms of palmitoylation and its functional implications in different types of tumors. We discuss how palmitoylation modulates key signaling pathways such as Ras and Wnt/β-catenin, influencing tumor cell behavior and the tumor microenvironment. Additionally, we examine the impact of palmitoylation on anti-tumor immunity and its potential as a therapeutic target. Understanding the intricate roles of palmitoylation in cancer biology not only advances our knowledge of tumor pathogenesis but also opens new avenues for targeted cancer therapies. Future research directions and clinical applications are also highlighted to guide the development of novel interventions.

## 1 Introduction

Palmitoylation, a reversible post-translational lipid modification, involves the covalent attachment of the 16-carbon saturated fatty acid palmitic acid to cysteine residues on target proteins via a thioester bond (1, 2). This process is catalyzed by specific palmitoyl acyltransferases (PATs), particularly the DHHC (Asp-His-His-Cys) family, named after its conserved catalytic motif (3, 4). The reversibility of palmitoylation stems from palmitoyl-protein thioesterases (PPTs), which cleave the thioester bond, enabling proteins to cycle between palmitoylated and depalmitoylated states (5). Unlike other lipid modifications such as myristoylation or prenylation, palmitoylation is dynamic, allowing precise temporal and spatial regulation of protein function in response to cellular signals (6).

Palmitoylation profoundly influences protein localization, trafficking, stability, and function (1, 7–9). By anchoring proteins to membranes, it critically determines the subcellular distribution of diverse proteins, including receptors, kinases, G-proteins, and ion channels (10, 11). Furthermore, this modification regulates protein-protein interactions, thereby modulating the assembly of signaling complexes and intracellular signal propagation (5, 12). The dynamic nature of palmitoylation enables cells to rapidly adapt to environmental cues and maintain homeostasis, establishing it as a crucial regulatory mechanism in various physiological processes.

In cancer, palmitoylation has emerged as a key player in tumorigenesis, impacting cancer initiation, progression, metastasis, and therapy resistance. Tumorigenesis is a multifaceted process driven by genetic mutations, epigenetic alterations, and aberrant signaling pathways that promote uncontrolled cell proliferation, survival, and invasion (13–15). Recent studies highlight palmitoylation’s role in modulating oncogene and tumor suppressor activity, influencing the balance between oncogenic and tumor-suppressive signaling pathways (16, 17). For example, the Ras family GTPases—frequently mutated in various cancers—undergo palmitoylation. This modification is essential for their proper plasma membrane localization and subsequent activation of downstream pathways such as MAPK/ERK and PI3K/AKT (18, 19). Palmitoylation dysregulation can lead to aberrant Ras localization and hyperactivation, promoting oncogenic transformation and tumor progression (20). Similarly, the Wnt/β-catenin signaling pathway—central to many cancers—is also regulated by palmitoylation. Palmitoylation of Wnt proteins is essential for their secretion and interaction with cell surface receptors, modulating β-catenin activation and downstream transcriptional programs that drive proliferation and differentiation (21).

Beyond regulating oncogenic signaling, palmitoylation shapes the tumor microenvironment and modulates anti-tumor immune responses (22). Tumors exploit palmitoylation to evade immune surveillance—for instance, by altering immune checkpoint proteins or cytokine receptor function. This immune modulation contributes to immune resistance, significantly challenging the efficacy of immunotherapies such as checkpoint inhibitors (17, 23). Understanding how palmitoylation regulates these processes could yield novel insights into overcoming immune resistance and improving cancer treatments.

Given the growing recognition of palmitoylation’s role in cancer, targeting this modification represents a promising therapeutic strategy. Small molecule inhibitors of PATs have shown efficacy in preclinical models, highlighting their potential as novel anticancer agents. This review comprehensively overviews the current understanding of palmitoylation in tumor biology, exploring its molecular mechanisms, functional impacts, and therapeutic potential. We also discuss challenges and future directions in studying cancer-associated palmitoylation, emphasizing opportunities for translating these findings into clinical applications.

## 2 Molecular mechanism of palmitoylation

Palmitoylation, a reversible post-translational lipid modification, involves the covalent attachment of palmitic acid (a 16-carbon saturated fatty acid) to cysteine residues on target proteins via a thioester bond (24, 25). This modification is catalyzed by palmitoyl acyltransferases (PATs), with the DHHC family (defined by its conserved Asp-His-His-Cys catalytic motif) being the most studied group (9, 26). The approximately 23 human DHHC enzymes, integral membrane proteins typically localized to the Golgi apparatus, endoplasmic reticulum (ER), and plasma membrane, exhibit distinct tissue distribution, subcellular localization, and substrate specificity (**Table 1**) (26). At these locations, DHHC PATs palmitoylate diverse protein substrates (27).

**Table 1:** Overview of DHHC family member functional and molecular characteristics.

DHHC Family Member	Function	Localization	Substrates	Ref
DHHC1 (ZDHHC1)	Primarily involved in cell signaling and growth regulation.	Golgi apparatus	Interacts mainly with Ras pathway-related proteins.	(28, 29)
DHHC2 (ZDHHC2)	Participates in synaptic plasticity, influencing neuronal activity.	Plasma membrane	Synaptic proteins like PSD-95.	(30–32)
DHHC3 (ZDHHC3, GODZ)	Closely associated with synaptic function in neurons.	Golgi apparatus	GABA receptors, PSD-95.	(32–34)
DHHC4 (ZDHHC4)	Function relatively unclear, but related to membrane protein localization.	Golgi apparatus and plasma membrane.	/	(35, 36)
DHHC5 (ZDHHC5)	Plays a role in cell migration, signal transduction, TNF-induced cell death and cellular homeostasis.	Plasma membrane	Involved with various receptors and membrane proteins.	(37–40)
DHHC6 (ZDHHC6)	Involved in protein folding, particularly in the endoplasmic reticulum.	Endoplasmic reticulum	Related to protein-folding proteins.	(41)
DHHC7 (ZDHHC7)	Involved in synaptic transmission and neurotransmitter release, , and also participates in pyroptosis of cells.	Golgi apparatus and plasma membrane	Neurotransmitter receptors and synaptic proteins.	(42–44)
DHHC8 (ZDHHC8)	Associated with neurodevelopmental disorders, including potential links to schizophrenia.	Golgi apparatus	NMDA receptor.	(45, 46)
DHHC9 (ZDHHC9)	Regulates the localization and function of Ras family small GTPases.	Golgi apparatus	H-Ras and N-Ras.	(47)
DHHC10 (ZDHHC10)	Function not fully understood, possibly related to mitochondrial function.	Mitochondria.	/	(48)
DHHC11 (ZDHHC11)	Less studied, with unclear function and substrate recognition.	/	/	^/^
DHHC12 (ZDHHC12)	Potentially involved in protein transport and localization, but not well studied.	/	/	(49)
DHHC13 (ZDHHC13, HIP14)	Linked to Huntington’s disease, regulating synaptic protein function.	Golgi apparatus and plasma membrane	Huntingtin protein.	(50, 51)
DHHC14 (ZDHHC14)	Function unclear, less studied.	/	/	/
DHHC15 (ZDHHC15)	Participates in cell signaling and protein localization.	Golgi apparatus and plasma membrane.	/	(52)
DHHC16 (ZDHHC16)	Plays a role in regulating post-translational modifications of proteins.	Golgi apparatus.	/	(53)
DHHC17 (ZDHHC17, HIP14L)	Associated with neurodegenerative diseases, affecting neuronal health.	Golgi apparatus and plasma membrane	Huntingtin protein and other neuro-related proteins.	(54)
DHHC18 (ZDHHC18)	Function unclear, less studied.	/	/	^/^
DHHC19 (ZDHHC19)	Less studied, possibly related to membrane protein localization.	/	/	(55)
DHHC20 (ZDHHC20)	Possibly related to immunity.	Golgi apparatus and plasma membrane.	/	(56)
DHHC21 (ZDHHC21)	Regulates inflammatory response and cell signaling.	Plasma membrane	Possibly involved with receptors and signaling molecules.	(57)
DHHC22 (ZDHHC22)	Function unclear, less studied.	/	/	/
DHHC23 (ZDHHC23)	Less studied, potentially related to certain diseases.	/	/	/
DHHC1 (ZDHHC1)	Primarily involved in cell signaling and growth regulation.	Golgi apparatus	Interacts mainly with Ras pathway-related proteins.	(28, 29)
DHHC2 (ZDHHC2)	Participates in synaptic plasticity, influencing neuronal activity.	Plasma membrane	Synaptic proteins like PSD-95.	(30–32)
DHHC3 (ZDHHC3, GODZ)	Closely associated with synaptic function in neurons.	Golgi apparatus	GABA receptors, PSD-95.	(32–34)
DHHC4 (ZDHHC4)	Function relatively unclear, but related to membrane protein localization.	Golgi apparatus and plasma membrane.	/	(35, 36)
DHHC5 (ZDHHC5)	Plays a role in cell migration and signal transduction.	Plasma membrane	Involved with various receptors and membrane proteins.	(37, 38)
DHHC6 (ZDHHC6)	Involved in protein folding, particularly in the endoplasmic reticulum.	Endoplasmic reticulum	Related to protein-folding proteins.	(39)
DHHC7 (ZDHHC7)	Involved in synaptic transmission and neurotransmitter release.	Golgi apparatus and plasma membrane	Neurotransmitter receptors and synaptic proteins.	(40, 41)
DHHC8 (ZDHHC8)	Associated with neurodevelopmental disorders, including potential links to schizophrenia.	Golgi apparatus	NMDA receptor.	(42, 43)
DHHC9 (ZDHHC9)	Regulates the localization and function of Ras family small GTPases.	Golgi apparatus	H-Ras and N-Ras.	(44)
DHHC10 (ZDHHC10)	Function not fully understood, possibly related to mitochondrial function.	Mitochondria.	/	(45)
DHHC11 (ZDHHC11)	Less studied, with unclear function and substrate recognition.	/	/	/
DHHC12 (ZDHHC12)	Potentially involved in protein transport and localization, but not well studied.	/	/	(46)
DHHC13 (ZDHHC13, HIP14)	Linked to Huntington’s disease, regulating synaptic protein function.	Golgi apparatus and plasma membrane	Huntingtin protein.	(47, 48)
DHHC14 (ZDHHC14)	Function unclear, less studied.	/	/	^/^
DHHC15 (ZDHHC15)	Participates in cell signaling and protein localization.	Golgi apparatus and plasma membrane.	/	(49)
DHHC16 (ZDHHC16)	Plays a role in regulating post-translational modifications of proteins.	Golgi apparatus.	/	(50)
DHHC17 (ZDHHC17, HIP14L)	Associated with neurodegenerative diseases, affecting neuronal health.	Golgi apparatus and plasma membrane	Huntingtin protein and other neuro-related proteins.	(51)
DHHC18 (ZDHHC18)	Function unclear, less studied.	/	/	/
DHHC19 (ZDHHC19)	Less studied, possibly related to membrane protein localization.	/	/	(52)
DHHC20 (ZDHHC20)	Possibly related to immunity.	Golgi apparatus and plasma membrane.	/	(53)
DHHC21 (ZDHHC21)	Regulates inflammatory response and cell signaling.	Plasma membrane	Possibly involved with receptors and signaling molecules.	(54)
DHHC22 (ZDHHC22)	Function unclear, less studied.	/	/	/
DHHC23 (ZDHHC23)	Less studied, potentially related to certain diseases.	/	/	/
DHHC1 (ZDHHC1)	Primarily involved in cell signaling and growth regulation.	Golgi apparatus	Interacts mainly with Ras pathway-related proteins.	(28, 29)
DHHC2 (ZDHHC2)	Participates in synaptic plasticity, influencing neuronal activity.	Plasma membrane	Synaptic proteins like PSD-95.	(30–32)
DHHC3 (ZDHHC3, GODZ)	Closely associated with synaptic function in neurons.	Golgi apparatus	GABA receptors, PSD-95.	(32–34)
DHHC4 (ZDHHC4)	Function relatively unclear, but related to membrane protein localization.	Golgi apparatus and plasma membrane.	/	(35, 36)
DHHC5 (ZDHHC5)	Plays a role in cell migration and signal transduction.	Plasma membrane	Involved with various receptors and membrane proteins.	(37, 38)
DHHC6 (ZDHHC6)	Involved in protein folding, particularly in the endoplasmic reticulum.	Endoplasmic reticulum	Related to protein-folding proteins.	(39)
DHHC7 (ZDHHC7)	Involved in synaptic transmission and neurotransmitter release.	Golgi apparatus and plasma membrane	Neurotransmitter receptors and synaptic proteins.	(40, 41)
DHHC8 (ZDHHC8)	Associated with neurodevelopmental disorders, including potential links to schizophrenia.	Golgi apparatus	NMDA receptor.	(42, 43)
DHHC9 (ZDHHC9)	Regulates the localization and function of Ras family small GTPases.	Golgi apparatus	H-Ras and N-Ras.	(44)
DHHC10 (ZDHHC10)	Function not fully understood, possibly related to mitochondrial function.	Mitochondria.	/	(45)
DHHC11 (ZDHHC11)	Less studied, with unclear function and substrate recognition.	/	/	/
DHHC12 (ZDHHC12)	Potentially involved in protein transport and localization, but not well studied.	/	/	(46)
DHHC13 (ZDHHC13, HIP14)	Linked to Huntington’s disease, regulating synaptic protein function.	Golgi apparatus and plasma membrane	Huntingtin protein.	(47, 48)
DHHC14 (ZDHHC14)	Function unclear, less studied.	/	/	^/^
DHHC15 (ZDHHC15)	Participates in cell signaling and protein localization.	Golgi apparatus and plasma membrane.	/	(49)
DHHC16 (ZDHHC16)	Plays a role in regulating post-translational modifications of proteins.	Golgi apparatus.	/	(50)
DHHC17 (ZDHHC17, HIP14L)	Associated with neurodegenerative diseases, affecting neuronal health.	Golgi apparatus and plasma membrane	Huntingtin protein and other neuro-related proteins.	(51)
DHHC18 (ZDHHC18)	Function unclear, less studied.	/	/	^/^
DHHC19 (ZDHHC19)	Less studied, possibly related to membrane protein localization.	/	/	(52)
DHHC20 (ZDHHC20)	Possibly related to immunity.	Golgi apparatus and plasma membrane.	/	(53)
DHHC21 (ZDHHC21)	Regulates inflammatory response and cell signaling.	Plasma membrane	Possibly involved with receptors and signaling molecules.	(54)
DHHC22 (ZDHHC22)	Function unclear, less studied.	/	/	^/^
DHHC23 (ZDHHC23)	Less studied, potentially related to certain diseases.	/	/	^/^

/, Not Applicable.

DHHC enzymes share common structural features, including multiple transmembrane domains (typically four to six) and a cytosolic catalytic domain containing the critical DHHC motif (58, 59). Their catalytic mechanism involves two key steps: autoacylation and substrate acylation (60–62). First, the enzyme undergoes autoacylation: a cysteine residue within the DHHC motif forms a thioester bond with palmitoyl-CoA, transferring the palmitoyl group to the enzyme itself. Subsequently, the palmitoyl group is transferred from the autoacylated DHHC enzyme to a cysteine residue on the substrate protein, facilitated by the close proximity of the substrate cysteine to the enzyme’s catalytic site, resulting in substrate palmitoylation.

Substrate recognition by DHHC enzymes is highly specific and governed by multiple factors: the amino acid sequence context surrounding the target cysteine, substrate localization, palmitoylation consensus motifs, and regulation by accessory proteins (37, 63–65). Specifically, the local amino acid sequence near the target cysteine critically determines recognition. Particular DHHC enzymes preferentially recognize substrates containing specific sequence motifs, often found near transmembrane domains or lipid-binding regions. Furthermore, the subcellular localization of both the enzyme and substrate is crucial for efficient palmitoylation. For example, substrates localized to the Golgi apparatus or plasma membrane are primarily palmitoylated by DHHC enzymes residing in those compartments (27). Although no universal palmitoylation consensus sequence exists, some DHHC enzymes exhibit preferences for substrates with specific motifs or structural features, such as a hydrophobic region preceding the cysteine or proximity to other lipid modifications like myristoylation or prenylation (27, 65). Finally, the activity and substrate specificity of DHHC enzymes can be regulated by accessory proteins or cofactors, which may recruit specific substrates or modulate enzyme localization and activity.

Protein palmitoylation is a reversible process enabling dynamic regulation of protein function. This reversibility occurs through palmitoyl-protein thioesterases (PPTs), such as PPT1 and PPT2, which hydrolyze the thioester bond to remove palmitoyl groups from substrate proteins (5, 66, 67). Depalmitoylation can occur in various cellular compartments, providing a mechanism for spatiotemporal control of palmitoylation. This dynamic nature allows cells to precisely modulate proteins involved in critical processes like signal transduction, membrane trafficking, and cell-cell communication (2, 68, 69). By regulating protein palmitoylation status, cells rapidly adapt to environmental changes, fine-tune signaling pathways, and maintain homeostasis (2, 70, 71). Such regulation is particularly vital for synaptic plasticity in neurons and immune responses across cell types.

Aberrant palmitoylation is implicated in diverse diseases, including cancer, neurodegenerative disorders, and infectious diseases (1, 72, 73). Dysregulation of DHHC enzymes or PPTs can cause mislocalization or dysfunction of key regulatory proteins, thereby driving disease pathogenesis. Given palmitoylation’s critical cellular roles, targeting its regulatory enzymes represents a promising therapeutic strategy. Small-molecule inhibitors against specific DHHC enzymes show potential in preclinical models, highlighting novel avenues for anticancer drug development.

In the following sections, we will further explore the role of palmitoylation in tumor progression.

## 3 Role of palmitoylation in tumors

This section examines how DHHC family members regulate tumor-associated proteins through palmitoylation. As key enzymes governing this modification, DHHC proteins control the palmitoylation status of critical oncoproteins and tumor suppressors, thereby dictating their activity, localization, and stability in cancer cells. We analyze how these regulatory mechanisms contribute to tumor initiation, progression, invasion, and metastasis. **Table 2** systematically summarizes current research findings on palmitoylated tumor-associated proteins, their functional roles, and associated DHHC enzymes.

**Table 2:** Regulation of palmitoylation in key tumor-associated proteins.

Protein name	Cancer species	Functions/mechanism	Clinical significance	Reference
CD36	Breast Cancer	CD36 deficiency induces ER stress while mitigating the pro-metastatic effects of a high-fat diet (HFD); only fully palmitoylated CD36 can rescue this effect.	CD36 supports HFD-driven tumor progression by preventing SFA-induced lipotoxicity.	(74)
KRAS4A	Pan-Cancer	The palmitoylation-depalmitoylation cycle of KRAS4A co-localizes it with HK1 on the mitochondrial outer membrane.	May lead to unique metabolic vulnerabilities, serving as a therapeutic target.	(75)
EpCAM-claudin-4 or -7-CD82 complex	Ovarian Cancer	Palmitoylation promotes complex formation.	Plays a significant role in ovarian cancer progression and metastasis.	(76)
CKAP4	Lung Cancer	CKAP4 regulates its release from lung cancer cells to exosomes via palmitoylation.	CKAP4 could be a key target for developing novel lung cancer therapeutic strategies.	(77)
RhoU	Prostate Cancer	RhoU can self-associate within cells, a process dependent on C-terminal palmitoylation.	Promotes prostate cancer progression.	(78)
KRAS4A	Leukemia	Mutation of KRAS4A palmitoylation sites significantly reduces its leukemogenic potential, with the KIKK motif being crucial for its transformation activity.	Disrupting the KIKK membrane-targeting motif may enhance therapeutic outcomes.	(79)
BTK-C	Epithelial Cancer	BTK-C is palmitoylated at two cysteine residues, regulating its plasma membrane localization in a PIP3-dependent manner.	Contributes to understanding kinase subtype regulation mechanisms, possibly providing new therapeutic targets.	(80)
CSC	Hepatocellular Carcinoma	Proteins in CSCs undergo palmitoylation after PA treatment, playing a critical role in CSC spheroid formation; palmitoylation inhibitors cerulenin and 2-bromopalmitate significantly reduce CSC spheroid formation capacity.	Palmitoylation plays a key role in regulating the tumor-initiating capacity of CSCs.	(81)
AQP4	Pan-Cancer	The structural stability of AQP4 is affected by ROS and palmitoylation, with the latter reducing its ROS transmission efficiency.	Provides a theoretical basis for developing new therapeutic strategies in combination with radiotherapy.	(82)
Cld7	Gastrointestinal Tumors	Palmitoylated cld7 is enriched in glycolipid-enriched membrane domain-derived TEX, which promote tumor cell migration, invasion, and (lymph)angiogenesis.	RTK inhibitors may be effective strategies for treating tumors driven by CIC-TEX.	(83)
FASN	Prostate Cancer	FASN also regulates cell adhesion and migration by affecting the palmitoylation of atypical GTPase RhoU.	Highlights the importance of FASN in regulating prostate cancer cell motility, supporting it as a potential therapeutic target.	(84)
Claudin7	Pan-Cancer	Claudin7 is palmitoylated in glycolipid-enriched membrane domains (GEM), a modification that promotes the integration of signaling molecules in GEM.	Highlights the potential significance of different Claudin7-derived microvesicles in therapeutic strategies.	(85)
STEAP1, STEAP2 and ABCC4	Prostate Cancer	STEAP1, STEAP2, and ABCC4 are identified as specific palmitoylated proteins, abundant in large EV (L-EV) and small EV (S-EV) of prostate cancer. Their localization in EVs decreases upon palmitoylation inhibition.	This post-translational modification may play a role in the sorting of EV-associated secretory proteome, potentially allowing for selective detection of disease biomarkers.	(86)
LPCAT1	Castration- Resistant Prostate Cancer (CRPC)	LPCAT1 promotes CRPC growth in an androgen-dependent manner through nuclear relocalization and histone H4 palmitoylation, increasing mRNA synthesis rates.	The results highlight LPCAT1's potential as a therapeutic target for CRPC.	(87)
KAI1/CD82	Metastatic Prostate Cancer (PC3)	Palmitoylation-deficient KAI1/CD82 mutants lead to the restoration of p130(CAS)-CrkII coupling, significantly reversing its inhibitory effect on PC3 cell migration and invasion.	The results emphasize the importance of palmitoylation in KAI1/CD82's role in inhibiting cancer cell migration and invasion.	(88)
Go	Pan-Cancer	Palmitoylation of Go enhances the internal interaction between Gαo and Gβγ, strengthens the coupling effect between Go and GPR97, stabilizes the ligand-binding pocket of GPR97, and enhances ligand affinity.	These findings provide new insights into the regulatory mechanisms of aGPCRs, guiding the future design of aGPCR-targeted drugs.	(89)
YKT6	Pancreatic Cancer	PVT1 promotes exosome secretion in PC cells by regulating YKT6 and VAMP3 colocalization and YKT6 palmitoylation, thereby facilitating MVB fusion with the plasma membrane.	These results deepen our understanding of the role of PVT1 in tumor biology.	(90)
NTSR-1	Breast Cancer	Dual palmitoylation of NTSR-1 at Cys381 and Cys383 is involved in regulating NTS-mediated ERK 1/2 phosphorylation.	Palmitoylation serves as a novel pharmacological target for inhibiting mitotic signaling of NTSR-1 in breast cancer cells.	(91)
2-ME	Lung Cancer	2-ME may interact with acyl protein thioesterase (APT1) as its inhibitor, enhancing protein palmitoylation and oxidative stress response in lung cancer cells.	2-ME may be a potential tumor biomarker in lung cancer progression and could be used as an adjunct or neoadjuvant therapy.	(92)
eIF3L	Prostate Cancer	Androgens significantly increase the palmitoylation level of eIF3L (a subunit of eIF3), promoting prostate cancer cell proliferation by enhancing translation rates.	Androgen-induced elevation of eIF3L levels may serve as an early biomarker for prostate cancer.	(93)
CD44	Breast Cancer	Point mutation at the palmitoylation site of CD44 reduces its raft affiliation in invasive breast cancer cells, increases CD44 co-precipitation with ezrin, thereby enhancing cell migration ability. Additionally, the palmitoylation-deficient CD44 mutant can induce epithelial-mesenchymal transition and increase cell motility.	Indicates that CD44 palmitoylation could be a new therapeutic target for breast cancer.	(94)
CKAP4	Pan-Cancer	CKAP4 binds to the mitochondrial outer membrane protein VDAC2 in a palmitoylation-dependent manner at Cys100.	CKAP4 plays an important role in maintaining mitochondrial function by regulating ER-mitochondria contact sites and VDAC2 binding, dependent on CKAP4 palmitoylation.	(95)
CD44	Hepatocellular Carcinoma	Inhibition of CD44 or its palmitoylation can abolish the inhibitory effect of cholesterol on HCC metastasis, preventing CD44 localization in lipid rafts.	Regulating CD44 localization in lipid rafts is a potential therapeutic strategy.	(96)
flotilin-1	Pan-Cancer	IGF-1-dependent depalmitoylation and repalmitoylation of flotillin-1 regulate the tyrosine kinase activation of plasma membrane-localized IGF-1R. When flotillin-1's palmitoylation function is impaired, preventing its turnover, cancer cell proliferation after IGF-1R signaling activation is eliminated.	Palmitoylation of flotillin-1 is a novel mechanism for regulating IGF-1R intracellular localization and activation.	(97)
ERα	Pan-Cancer	Palmitoylation of ERα promotes its membrane association and interaction with membrane protein Caveolin-1, influencing non-genomic activities like signal pathway activation and cell proliferation. E2 dose and time-dependently reduce ERα palmitoylation and its interaction with Caveolin-1.	These findings reveal the physiological role of ERα palmitoylation in regulating cell membrane receptor localization and E2-induced cell proliferation.	(98)
AR8	Prostate Cancer	AR8 lacks a DNA-binding domain and primarily localizes to the plasma membrane through palmitoylation of two cysteine residues within its unique C-terminal sequence.	Membrane-associated AR8 may contribute to the development of castration resistance in prostate cancer by enhancing AR-mediated hormone and growth factor responses.	(99)
TMX1	Pan-Cancer	TMX1 targets the MAM via its thioredoxin motif and palmitoylation, influencing ER-mitochondria contact, which in turn affects bioenergetic supply and accelerates tumor growth.	TMX1, as a thiol-based tumor suppressor, may function by increasing mitochondrial ATP production and promoting the apoptotic process.	(100)
ERbeta	Pan-Cancer	Palmitoylation is essential for ERbeta's localization to the plasma membrane, facilitating its interaction with Caveolin-1 and involvement in rapid signaling pathways related to cell proliferation.	Palmitoylation is part of the molecular mechanism of ERbeta, allowing these receptors to interact with other proteins at the plasma membrane.	(101)
SCP1	Pan-Cancer	The plasma membrane localization of SCP1 is regulated by the palmitoylation of a conserved cysteine motif at its NH2-terminus, which is crucial for inhibiting angiogenesis and tumor growth.	These results reveal a novel mechanism by which SCP1 shuttles between the nucleus and the plasma membrane.	(102)
NADA	Pan-Cancer	NADA inhibits the membrane translocation and tumorigenic transformation of oncogenic KRAS4A, and it redistributes cytoplasmic NRAS to the Golgi apparatus in a palmitoylation-dependent manner.	These findings provide crucial insights for the development of novel targeted therapies for various human cancers.	(103)
Gα13	Pan-Cancer	Overexpression of wild-type Gα13 significantly enhances serum response factor (SRF)-mediated transcriptional activity, partly through S-palmitoylation modifications.	These findings deepen our understanding of Gα13's role in promoting tumor growth and oncogenic signaling pathways.	(104)
E2	Colorectal Cancer	In DLD-1 colorectal cancer cells, E2 induces rapid translation of ERβ mRNA and late-phase transcriptional enhancement, both of which depend on E2-induced persistent and palmitoylation-dependent p38/MAPK activation.	These data suggest that rapid signaling pathways exert fine-tuned control over the protective effects of E2 against colorectal cancer growth.	(105)
P1MK5E	Triple-negative Breast Cancer	The N-terminal palmitoylated magainin derivative (P1MK5E) exhibits strong cytotoxicity by enhancing its turn structure motif.	It holds potential advantages for targeting apoptosis resistance pathways in triple-negative breast cancer cells.	(106)
SR	Breast Cancer	Heat shock protein 27 (Hsp27) enhances the palmitoylation of SR by binding to estrogen receptor α (ERα), thereby increasing the interaction between ERα and Caveolin-1. This process promotes membrane localization, kinase activation, and DNA synthesis in breast cancer cells.	This reveals its potential role in tumor biology and suggests it could become a new target for treating hormone-responsive cancers.	(107)
H-RAS	Cervical Cancer	H-rev107 forms a complex with H-RAS and reduces the palmitoylation level of H-RAS. In HtTA cervical cancer cells, H-rev107 lowers the levels of activated RAS (RAS-GTP) and decreases ELK1-mediated transactivation.	These results deepen the understanding of H-rev107's mechanism in regulating H-RAS activity.	(108)
CDCP1	Kaposi's Sarcoma (KS)	vIRF1 also regulates the ubiquitin-proteasome pathway to degrade the metastasis suppressor CD82, thereby protecting CDCP1 from CD82-mediated palmitoylation-dependent degradation. The activation of CDCP1 further stimulates the AKT signaling pathway, playing a crucial role in vIRF1-induced cell motility.	These findings reveal the critical role of vIRF1 in the pathogenesis of Kaposi's sarcoma, providing potential pathways for the development of new therapeutic targets.	(109)
YES	Colorectal Cancer	The oncogenic signaling of YES depends on the palmitoylation of its SH4 domain, which regulates YES localization in cholesterol-rich membrane microdomains.	These results elucidate the mechanism by which YES functions in CRC cells.	(110)
Rab38	Melanoma	NY-MEL-1 encodes a novel rabbit GTPase, Rab38, which features a unique COOH terminus that allows for post-translational translation and palmitoylation modifications. This is relatively rare among other Rab proteins and is typically observed in Ras proteins.	Rab38 may serve as a novel biomarker and potential prognostic indicator for melanoma and other malignancies.	(111)
SLC7A11	Hepatocellular Carcinoma	DUXAP8 acts on SLC7A11 to promote its palmitoylation and prevent its lysosomal degradation, thereby reducing the sensitivity of HCC cells to sorafenib-induced ferroptosis.	Combining sorafenib with DUXAP8 silencing may overcome resistance and improve therapeutic outcomes for patients with advanced HCC.	(112)
GARS1	Pan-Cancer	GARS1 is secreted via extracellular vesicles (EVs) with a diameter of approximately 20-58 nm, anchored on their surface by palmitoylation of the C390 residue.	These results suggest potential applications of GARS1 through specific secretory vesicles in cancer therapy.	(113)
PAR2	Pan-Cancer	PAR2 undergoes palmitoylation at cysteine 361, which is crucial for its intracellular trafficking and efficient cell surface localization.	The results reveal that palmitoylation is a key factor in maintaining the lifecycle and function of PAR2.	(114)
Smad3	Glioblastoma	Palmitoylation of Smad3 mediated by the palmitoyltransferase ZDHHC19, however, promotes the activation of the TGF-β signaling pathway. Moreover, its interaction with EP300 enhances the expression of mesenchymal markers in the mesenchymal subtype of GBM.	These findings suggest that Smad3 could be a key target for the treatment of gliomas.	(115)
Transferrin Receptor-1	Pan-Cancer	DHA induces palmitoylation of transferrin receptor 1 and co-localizes with Caveolin-1. Cyclosporin A reverses the effects of DHA on cell cycle and apoptosis-related genes, while siRNA-mediated downregulation of transferrin receptor 1 effectively reduces cell sensitivity to DHA.	These findings reveal that DHA combats cancer by regulating transferrin receptor 1 through a non-classical endocytosis pathway.	(116)
GSDME	Pan-Cancer	In chemotherapy-induced pyroptosis, the C-terminus of GSDME (GSDME-C) undergoes palmitoylation, and 2-bromopalmitate (2-BP) inhibits this palmitoylation and subsequent pyroptosis. Mutations at the palmitoylation sites on GSDME also reduce chemotherapy-induced pyroptosis.	These findings provide new targets for shifting between chemotherapy-induced pyroptosis and apoptosis.	(117)
α-tubulin	Prostate Cancer	Androgen treatment significantly enhances the palmitoylation levels of α-tubulin and Rab7a, modifications that are critical for cell proliferation.	These palmitoylation modifications may serve as potential biomarkers for early-stage prostate cancer.	(118)
CD36	Breast Cancer	CD36 deficiency induces ER stress while mitigating the pro-metastatic effects of a high-fat diet (HFD); only fully palmitoylated CD36 can rescue this effect.	CD36 supports HFD-driven tumor progression by preventing SFA-induced lipotoxicity.	(71)
KRAS4A	Pan-Cancer	The palmitoylation-depalmitoylation cycle of KRAS4A co-localizes it with HK1 on the mitochondrial outer membrane.	May lead to unique metabolic vulnerabilities, serving as a therapeutic target.	(72)
EpCAM-claudin-4 or -7-CD82 complex	Ovarian Cancer	Palmitoylation promotes complex formation.	Plays a significant role in ovarian cancer progression and metastasis.	(73)
CKAP4	Lung Cancer	CKAP4 regulates its release from lung cancer cells to exosomes via palmitoylation.	CKAP4 could be a key target for developing novel lung cancer therapeutic strategies.	(74)
RhoU	Prostate Cancer	RhoU can self-associate within cells, a process dependent on C-terminal palmitoylation.	Promotes prostate cancer progression.	(75)
KRAS4A	Leukemia	Mutation of KRAS4A palmitoylation sites significantly reduces its leukemogenic potential, with the KIKK motif being crucial for its transformation activity.	Disrupting the KIKK membrane-targeting motif may enhance therapeutic outcomes.	(76)
BTK-C	Epithelial Cancer	BTK-C is palmitoylated at two cysteine residues, regulating its plasma membrane localization in a PIP3-dependent manner.	Contributes to understanding kinase subtype regulation mechanisms, possibly providing new therapeutic targets.	(77)
CSC	Hepatocellular Carcinoma	Proteins in CSCs undergo palmitoylation after PA treatment, playing a critical role in CSC spheroid formation; palmitoylation inhibitors cerulenin and 2-bromopalmitate significantly reduce CSC spheroid formation capacity.	Palmitoylation plays a key role in regulating the tumor-initiating capacity of CSCs.	(78)
AQP4	Pan-Cancer	The structural stability of AQP4 is affected by ROS and palmitoylation, with the latter reducing its ROS transmission efficiency.	Provides a theoretical basis for developing new therapeutic strategies in combination with radiotherapy.	(79)
Cld7	Gastrointestinal Tumors	Palmitoylated cld7 is enriched in glycolipid-enriched membrane domain-derived TEX, which promote tumor cell migration, invasion, and (lymph)angiogenesis.	RTK inhibitors may be effective strategies for treating tumors driven by CIC-TEX.	(80)
FASN	Prostate Cancer	FASN also regulates cell adhesion and migration by affecting the palmitoylation of atypical GTPase RhoU.	Highlights the importance of FASN in regulating prostate cancer cell motility, supporting it as a potential therapeutic target.	(81)
Claudin7	Pan-Cancer	Claudin7 is palmitoylated in glycolipid-enriched membrane domains (GEM), a modification that promotes the integration of signaling molecules in GEM.	Highlights the potential significance of different Claudin7-derived microvesicles in therapeutic strategies.	(82)
STEAP1, STEAP2 and ABCC4	Prostate Cancer	STEAP1, STEAP2, and ABCC4 are identified as specific palmitoylated proteins, abundant in large EV (L-EV) and small EV (S-EV) of prostate cancer. Their localization in EVs decreases upon palmitoylation inhibition.	This post-translational modification may play a role in the sorting of EV-associated secretory proteome, potentially allowing for selective detection of disease biomarkers.	(83)
LPCAT1	Castration- Resistant Prostate Cancer (CRPC)	LPCAT1 promotes CRPC growth in an androgen-dependent manner through nuclear relocalization and histone H4 palmitoylation, increasing mRNA synthesis rates.	The results highlight LPCAT1's potential as a therapeutic target for CRPC.	(84)
KAI1/CD82	Metastatic Prostate Cancer (PC3)	Palmitoylation-deficient KAI1/CD82 mutants lead to the restoration of p130(CAS)-CrkII coupling, significantly reversing its inhibitory effect on PC3 cell migration and invasion.	The results emphasize the importance of palmitoylation in KAI1/CD82's role in inhibiting cancer cell migration and invasion.	(85)
Go	Pan-Cancer	Palmitoylation of Go enhances the internal interaction between Gαo and Gβγ, strengthens the coupling effect between Go and GPR97, stabilizes the ligand-binding pocket of GPR97, and enhances ligand affinity.	These findings provide new insights into the regulatory mechanisms of aGPCRs, guiding the future design of aGPCR-targeted drugs.	(86)
YKT6	Pancreatic Cancer	PVT1 promotes exosome secretion in PC cells by regulating YKT6 and VAMP3 colocalization and YKT6 palmitoylation, thereby facilitating MVB fusion with the plasma membrane.	These results deepen our understanding of the role of PVT1 in tumor biology.	(87)
NTSR-1	Breast Cancer	Dual palmitoylation of NTSR-1 at Cys381 and Cys383 is involved in regulating NTS-mediated ERK 1/2 phosphorylation.	Palmitoylation serves as a novel pharmacological target for inhibiting mitotic signaling of NTSR-1 in breast cancer cells.	(88)
2-ME	Lung Cancer	2-ME may interact with acyl protein thioesterase (APT1) as its inhibitor, enhancing protein palmitoylation and oxidative stress response in lung cancer cells.	2-ME may be a potential tumor biomarker in lung cancer progression and could be used as an adjunct or neoadjuvant therapy.	(89)
eIF3L	Prostate Cancer	Androgens significantly increase the palmitoylation level of eIF3L (a subunit of eIF3), promoting prostate cancer cell proliferation by enhancing translation rates.	Androgen-induced elevation of eIF3L levels may serve as an early biomarker for prostate cancer.	(90)
CD44	Breast Cancer	Point mutation at the palmitoylation site of CD44 reduces its raft affiliation in invasive breast cancer cells, increases CD44 co-precipitation with ezrin, thereby enhancing cell migration ability. Additionally, the palmitoylation-deficient CD44 mutant can induce epithelial-mesenchymal transition and increase cell motility.	Indicates that CD44 palmitoylation could be a new therapeutic target for breast cancer.	(91)
CKAP4	Pan-Cancer	CKAP4 binds to the mitochondrial outer membrane protein VDAC2 in a palmitoylation-dependent manner at Cys100.	CKAP4 plays an important role in maintaining mitochondrial function by regulating ER-mitochondria contact sites and VDAC2 binding, dependent on CKAP4 palmitoylation.	(92)
CD44	Hepatocellular Carcinoma	Inhibition of CD44 or its palmitoylation can abolish the inhibitory effect of cholesterol on HCC metastasis, preventing CD44 localization in lipid rafts.	Regulating CD44 localization in lipid rafts is a potential therapeutic strategy.	(93)
flotilin-1	Pan-Cancer	IGF-1-dependent depalmitoylation and repalmitoylation of flotillin-1 regulate the tyrosine kinase activation of plasma membrane-localized IGF-1R. When flotillin-1's palmitoylation function is impaired, preventing its turnover, cancer cell proliferation after IGF-1R signaling activation is eliminated.	Palmitoylation of flotillin-1 is a novel mechanism for regulating IGF-1R intracellular localization and activation.	(94)
ERα	Pan-Cancer	Palmitoylation of ERα promotes its membrane association and interaction with membrane protein Caveolin-1, influencing non-genomic activities like signal pathway activation and cell proliferation. E2 dose and time-dependently reduce ERα palmitoylation and its interaction with Caveolin-1.	These findings reveal the physiological role of ERα palmitoylation in regulating cell membrane receptor localization and E2-induced cell proliferation.	(95)
AR8	Prostate Cancer	AR8 lacks a DNA-binding domain and primarily localizes to the plasma membrane through palmitoylation of two cysteine residues within its unique C-terminal sequence.	Membrane-associated AR8 may contribute to the development of castration resistance in prostate cancer by enhancing AR-mediated hormone and growth factor responses.	(96)
TMX1	Pan-Cancer	TMX1 targets the MAM via its thioredoxin motif and palmitoylation, influencing ER-mitochondria contact, which in turn affects bioenergetic supply and accelerates tumor growth.	TMX1, as a thiol-based tumor suppressor, may function by increasing mitochondrial ATP production and promoting the apoptotic process.	(97)
ERbeta	Pan-Cancer	Palmitoylation is essential for ERbeta's localization to the plasma membrane, facilitating its interaction with Caveolin-1 and involvement in rapid signaling pathways related to cell proliferation.	Palmitoylation is part of the molecular mechanism of ERbeta, allowing these receptors to interact with other proteins at the plasma membrane.	(98)
SCP1	Pan-Cancer	The plasma membrane localization of SCP1 is regulated by the palmitoylation of a conserved cysteine motif at its NH2-terminus, which is crucial for inhibiting angiogenesis and tumor growth.	These results reveal a novel mechanism by which SCP1 shuttles between the nucleus and the plasma membrane.	(99)
NADA	Pan-Cancer	NADA inhibits the membrane translocation and tumorigenic transformation of oncogenic KRAS4A, and it redistributes cytoplasmic NRAS to the Golgi apparatus in a palmitoylation-dependent manner.	These findings provide crucial insights for the development of novel targeted therapies for various human cancers.	(100)
Gα13	Pan-Cancer	Overexpression of wild-type Gα13 significantly enhances serum response factor (SRF)-mediated transcriptional activity, partly through S-palmitoylation modifications.	These findings deepen our understanding of Gα13's role in promoting tumor growth and oncogenic signaling pathways.	(101)
E2	Colorectal Cancer	In DLD-1 colorectal cancer cells, E2 induces rapid translation of ERβ mRNA and late-phase transcriptional enhancement, both of which depend on E2-induced persistent and palmitoylation-dependent p38/MAPK activation.	These data suggest that rapid signaling pathways exert fine-tuned control over the protective effects of E2 against colorectal cancer growth.	(102)
P1MK5E	Triple-negative Breast Cancer	The N-terminal palmitoylated magainin derivative (P1MK5E) exhibits strong cytotoxicity by enhancing its turn structure motif.	It holds potential advantages for targeting apoptosis resistance pathways in triple-negative breast cancer cells.	(103)
SR	Breast Cancer	Heat shock protein 27 (Hsp27) enhances the palmitoylation of SR by binding to estrogen receptor α (ERα), thereby increasing the interaction between ERα and Caveolin-1. This process promotes membrane localization, kinase activation, and DNA synthesis in breast cancer cells.	This reveals its potential role in tumor biology and suggests it could become a new target for treating hormone-responsive cancers.	(104)
H-RAS	Cervical Cancer	H-rev107 forms a complex with H-RAS and reduces the palmitoylation level of H-RAS. In HtTA cervical cancer cells, H-rev107 lowers the levels of activated RAS (RAS-GTP) and decreases ELK1-mediated transactivation.	These results deepen the understanding of H-rev107's mechanism in regulating H-RAS activity.	(105)
CDCP1	Kaposi's Sarcoma (KS)	vIRF1 also regulates the ubiquitin-proteasome pathway to degrade the metastasis suppressor CD82, thereby protecting CDCP1 from CD82-mediated palmitoylation-dependent degradation. The activation of CDCP1 further stimulates the AKT signaling pathway, playing a crucial role in vIRF1-induced cell motility.	These findings reveal the critical role of vIRF1 in the pathogenesis of Kaposi's sarcoma, providing potential pathways for the development of new therapeutic targets.	(106)
YES	Colorectal Cancer	The oncogenic signaling of YES depends on the palmitoylation of its SH4 domain, which regulates YES localization in cholesterol-rich membrane microdomains.	These results elucidate the mechanism by which YES functions in CRC cells.	(107)
Rab38	Melanoma	NY-MEL-1 encodes a novel rabbit GTPase, Rab38, which features a unique COOH terminus that allows for post-translational translation and palmitoylation modifications. This is relatively rare among other Rab proteins and is typically observed in Ras proteins.	Rab38 may serve as a novel biomarker and potential prognostic indicator for melanoma and other malignancies.	(108)
SLC7A11	Hepatocellular Carcinoma	DUXAP8 acts on SLC7A11 to promote its palmitoylation and prevent its lysosomal degradation, thereby reducing the sensitivity of HCC cells to sorafenib-induced ferroptosis.	Combining sorafenib with DUXAP8 silencing may overcome resistance and improve therapeutic outcomes for patients with advanced HCC.	(109)
GARS1	Pan-Cancer	GARS1 is secreted via extracellular vesicles (EVs) with a diameter of approximately 20-58 nm, anchored on their surface by palmitoylation of the C390 residue.	These results suggest potential applications of GARS1 through specific secretory vesicles in cancer therapy.	(110)
PAR2	Pan-Cancer	PAR2 undergoes palmitoylation at cysteine 361, which is crucial for its intracellular trafficking and efficient cell surface localization.	The results reveal that palmitoylation is a key factor in maintaining the lifecycle and function of PAR2.	(111)
Smad3	Glioblastoma	Palmitoylation of Smad3 mediated by the palmitoyltransferase ZDHHC19, however, promotes the activation of the TGF-β signaling pathway. Moreover, its interaction with EP300 enhances the expression of mesenchymal markers in the mesenchymal subtype of GBM.	These findings suggest that Smad3 could be a key target for the treatment of gliomas.	(112)
Transferrin Receptor-1	Pan-Cancer	DHA induces palmitoylation of transferrin receptor 1 and co-localizes with Caveolin-1. Cyclosporin A reverses the effects of DHA on cell cycle and apoptosis-related genes, while siRNA-mediated downregulation of transferrin receptor 1 effectively reduces cell sensitivity to DHA.	These findings reveal that DHA combats cancer by regulating transferrin receptor 1 through a non-classical endocytosis pathway.	(113)
GSDME	Pan-Cancer	In chemotherapy-induced pyroptosis, the C-terminus of GSDME (GSDME-C) undergoes palmitoylation, and 2-bromopalmitate (2-BP) inhibits this palmitoylation and subsequent pyroptosis. Mutations at the palmitoylation sites on GSDME also reduce chemotherapy-induced pyroptosis.	These findings provide new targets for shifting between chemotherapy-induced pyroptosis and apoptosis.	(114)
α-tubulin	Prostate Cancer	Androgen treatment significantly enhances the palmitoylation levels of α-tubulin and Rab7a, modifications that are critical for cell proliferation.	These palmitoylation modifications may serve as potential biomarkers for early-stage prostate cancer.	(115)

### 3.1 Pro-oncogenic ZDHHCs

A subset of ZDHHC enzymes, such as ZDHHC3, ZDHHC5, ZDHHC9, and ZDHHC20, function as potent oncogenic amplifiers by strategically palmitoylating key effector proteins that anchor to the plasma membrane. This spatial redistribution constitutively activates Ras/MAPK, Wnt/β-catenin, and PI3K/AKT pathways—core signaling cascades implicated in tumor proliferation, invasion, and therapy resistance. Critically, their overexpression or genetic amplification correlates with advanced TNM staging, metastatic recurrence, and poor overall survival (OS). Targeting these enzymes thus represents a promising strategy to dismantle oncogenic signaling scaffolds at their membrane-centric origins, particularly in cancers with defined pathway dependencies.

**Figure 1:**
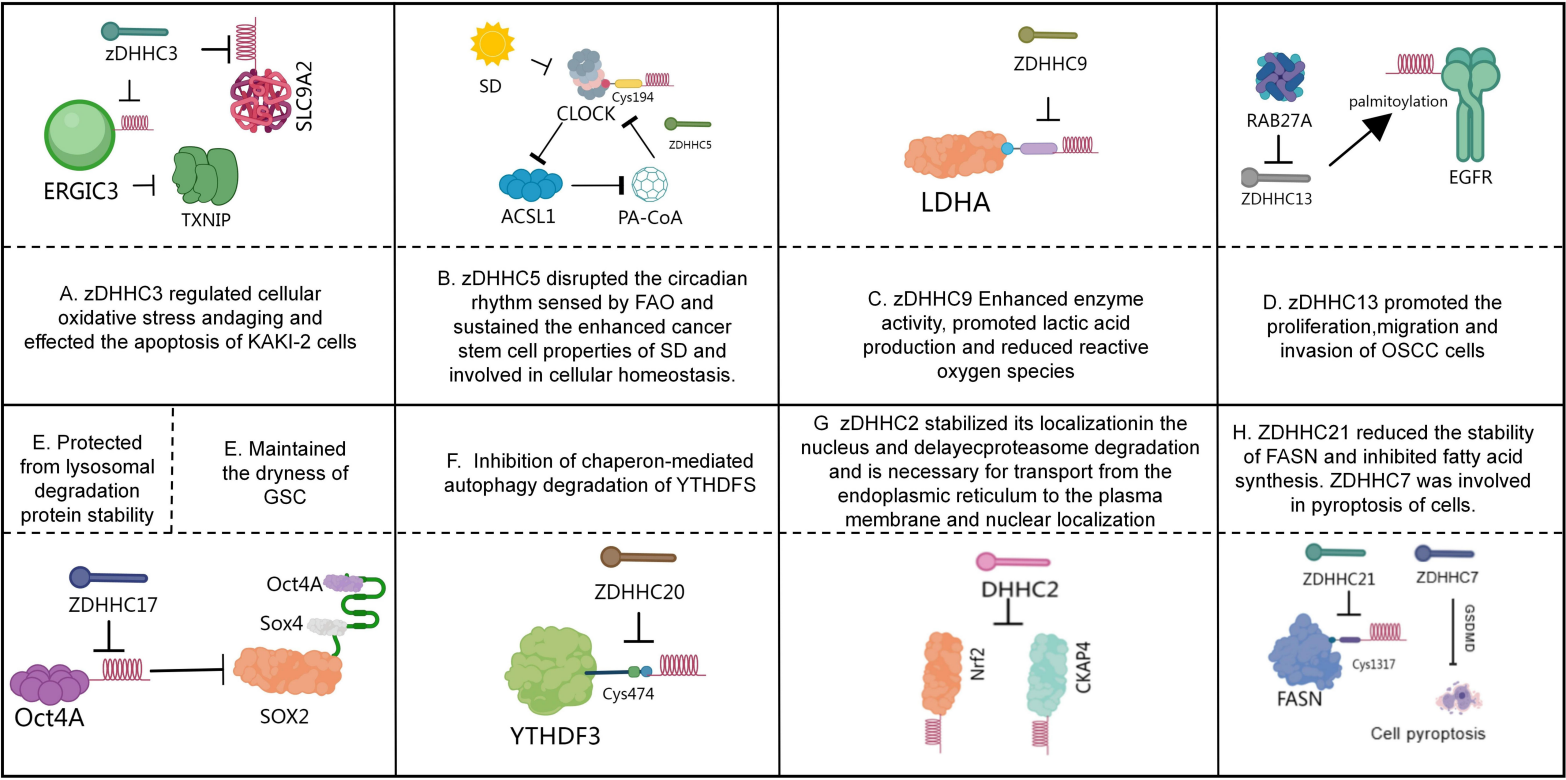
Role of palmitoylation in tumors. **(A)** The reduced DHHC3-dependent palmitoylation of ERGIC3 protein may play a crucial role in the upregulation of TXNIP (119). zDHHC3 influences the apoptosis process of Caki-2 cells by regulating the S-palmitoylation and expression levels of SLC9A2 (121). **(B)** CLOCK, dysregulated by SD, hyperactivates ACSL1 to produce PA-CoA, which promotes the S-palmitoylation of CLOCK-Cys194 in a ZDHHC5-dependent manner. This forms a positive feedback loop, preventing CLOCK degradation, leading to FAO-sensing circadian rhythm disruption, and maintaining SD-enhanced cancer stemness (126). **(C)** LDHA undergoes post-translational palmitoylation at Cysteine 163 by ZDHHC9, thereby enhancing its enzymatic activity, promoting lactate production, and reducing reactive oxygen species (ROS) production (128). **(D)** RAB27A may influence disease progression by regulating the palmitoylation of epidermal growth factor receptor (EGFR) mediated by zinc-finger DHHC-type 13 (ZDHHC13) (131). **(E)** ZDHHC17-mediated palmitoylation of Oct4A maintains GSC function by protecting Oct4A from lysosomal degradation and promoting its integration with Sox4 at the SOX2 enhancer region (132). **(F)** ZDHHC20 inhibits the autophagic degradation of YTHDF3 through S-palmitoylation at Cys474, leading to abnormal accumulation of MYC and promoting pancreatic cancer progression (136). **(G)** DHHC2 mediates the palmitoylation of Nrf2 in the cytoplasm, stabilizing its nuclear localization and delaying its proteasomal degradation (140). DHHC2-mediated palmitoylation of CKAP4 is essential for its transport from the endoplasmic reticulum to the plasma membrane and nuclear localization (142). **(H)** ZDHHC21 reduced the stability of FASN and inhibited fatty acid synthesis. ZDHHC7 was involved in pyroptosis of cells (145).

IIn metastatic breast cancer, DHHC3 overexpression correlates with reduced patient survival. Elimination of DHHC3 in xenograft models reduces primary tumor growth and lung metastasis while increasing oxidative stress, cellular senescence, and recruitment of anti-tumor immune cells (e.g., macrophages, NK cells). DHHC3 depletion also downregulates the oxidative stress regulator TXNIP, indicating DHHC3 promotes tumorigenesis by modulating oxidative stress and senescence pathways (**Figure 1A**) (119). Notably, curcumin selectively inhibits DHHC3 auto-palmitoylation, reducing ITGβ4 palmitoylation and suppressing breast cancer invasion without broadly disrupting cysteine modifications (120). Contrastingly, zDHHC3 is downregulated in kidney renal clear cell carcinoma (KIRC), where high expression correlates with favorable prognosis. In KIRC, zDHHC3 regulates apoptosis through SLC9A2 palmitoylation and expression (**Figure 1A**) (121). ZDHHC5 demonstrates tissue-specific oncogenicity: In lung adenocarcinoma (LUAD), ZDHHC5 overexpression correlates with tumor progression and INCENP expression. Nuclear-localized ZDHHC5 modulates cancer stem cells (CSCs) via INCENP palmitoylation at Cys15 (122). In pancreatic cancer, ZDHHC5-mediated palmitoylation of SSTR5 enables cancer cell proliferation. The inhibitor lomitapide blocks this process, enhancing anti-tumor responses (123). In gliomas, mutant p53 cooperates with NF-Y to upregulate ZDHHC5, promoting stemness and tumorigenicity through EZH2 palmitoylation/phosphorylation (124). In NSCLC, DHHC5 silencing inhibits proliferation, colony formation, invasion, and xenograft growth without affecting normal bronchial cells (38). In triple-negative breast cancer (TNBC), ZDHHC5 palmitoylates Flotillin-1, stabilizing it to drive metastasis. Flotillin-1 palmitoylation deficiency or pharmacological inhibition suppresses tumor progression and lung metastasis (125).

Interestingly, sleep deprivation (SD) disrupts circadian rhythms by increasing palmitoyl-CoA (PA-CoA) synthesis catalyzed by ACSL1. SD-induced CLOCK hyperactivation elevates PA-CoA levels via ACSL1 and promotes ZDHHC5-dependent palmitoylation of CLOCK at Cys194. This forms a positive feedback loop that stabilizes CLOCK, preventing its degradation and perpetuating circadian disruption while maintaining SD-enhanced cancer stemness. Timely β-endorphin supplementation resets circadian rhythms and suppresses Acsl1 expression, mitigating SD-driven tumorigenesis. Clinically, poor sleep quality and low serum β-endorphin correlate with tumor progression and elevated CLOCK/ACSL1 expression, suggesting β-endorphin as a potential therapeutic for SD-associated cancers (**Figure 1B**) (126). Additionally, DHHC5 binds and palmitoylates the auxiliary protein Golga7b, stabilizing it at the plasma membrane and blocking clathrin-mediated endocytosis. The DHHC5/Golga7b complex recruits adhesion proteins (e.g., plakophilin-3, desmoplakin-2) to desmosomes, with desmoplakin-2 localization being critically dependent on this complex. Disruption of DHHC5/Golga7b impairs cell adhesion, underscoring their essential role in intercellular cohesion (127). Collectively, these findings position DHHC5 as a novel therapeutic target in cancer, providing a mechanistic foundation for developing targeted anticancer strategies.

In non-tumor diseases, studies have shown that TNF-induced palmitoylation of RIPK1 (mediated by DHHC5 and dependent on its K63 ubiquitination) is a key mechanism that activates RIPK1 kinase activity and bypasses the cell death checkpoint, thereby inducing downstream apoptosis/necroptosis. This modification enhances the activity by promoting homophilic interactions in the kinase domain of RIPK1. Under pathological conditions such as MASH, fatty acid-driven DHHC5 increase promotes RIPK1 palmitoylation and cytotoxicity, highlighting the importance of this pathway in inflammatory diseases and providing new target ideas for therapeutic intervention (40). In addition, palmitoylation (especially the Beclin 1 modification mediated by DHHC5) plays a crucial role in regulating autophagy initiation and maintaining cellular protein homeostasis. DHHC5-mediated Beclin 1 S-palmitoylation drives the assembly and activation of the functional PI3KC3-C1 complex by promoting its hydrophobic interaction with ATG14L and VPS15. The downregulation of DHHC5 expression during aging leads to a reduction in Beclin 1 palmitoylation, becoming an important driving factor for the decline in autophagy function. This decline is manifested as protein homeostasis collapse within neurons and exacerbation of neurodegeneration in mouse models of Alzheimer’s disease, emphasizing the importance of the DHHC5-Beclin1 pathway in maintaining cellular homeostasis in aging-related diseases (especially neurodegenerative diseases), and providing potential targets for intervention (such as targeting DHHC5 or Beclin1 palmitoylation) (39).

ZDHHC9 overexpression in pancreatic cancer correlates with poor prognosis. It palmitoylates lactate dehydrogenase A (LDHA)—a key glycolytic enzyme regulating the Warburg effect—at Cys163. This modification enhances LDHA enzymatic activity, promoting lactate production and reducing reactive oxygen species (ROS). Substituting endogenous LDHA with a palmitoylation-deficient mutant suppresses pancreatic cancer proliferation, increases tumor-infiltrating T cells, limits tumor growth, and alters chemotherapy response. Notably, ZDHHC9-mediated LDHA palmitoylation is upregulated in gemcitabine-resistant pancreatic cancer (**Figure 1C**) (128). Additionally, ZDHHC9 palmitoylates glucose transporter GLUT1 at Cys207, maintaining its plasma membrane localization. This modification sustains glycolytic flux, proliferation, colony formation, and tumorigenesis in glioblastoma (129). ZDHHC12 mediates CLDN3 palmitoylation at three juxtamembrane cysteine residues, critical for ovarian cancer progression. Palmitoylation stabilizes CLDN3 and ensures its proper membrane localization. Loss of palmitoylation abolishes CLDN3’s oncogenic effects. Knocking down ZDHHC12 disrupts CLDN3 membrane targeting/stability and impairs ovarian cancer tumorigenicity, highlighting ZDHHC12 as a therapeutic target (130). In oral squamous cell carcinoma (OSCC), RAB27A overexpression correlates with metastasis and poor survival. RAB27A regulates ZDHHC13-mediated EGFR palmitoylation. Silencing RAB27A significantly inhibits OSCC proliferation, migration, and invasion (**Figure 1D**) (131).

ZDHHC17 palmitoylates Oct4A in glioblastoma stem cells (GSCs), maintaining its stability and stemness. Palmitoylation prevents Oct4A lysosomal degradation and facilitates its interaction with Sox4 at the SOX2 enhancer. Oct4A palmitoylation inhibitors effectively suppress GSC self-renewal and tumorigenicity, revealing a promising therapeutic strategy (**Figure 1E**) (132). Palmoylation precisely and hierarchically regulates the NLRP3 inflammasome at different activation stages by targeting multiple conserved cysteine sites on the NLRP3 protein (such as Cys130/901/958, Cys837/838, Cys419, Cys844, Cys8). In the priming stage (initiation), the palmitoylation catalyzed by DHHC1/3/5/7 (and DHHC1 catalyzing Cys958) promotes the localization of NLRP3 to the Golgi network (TGN), laying a spatial foundation for subsequent activation, and this process is negatively regulated by the thioesterase APT2. In the activation stage, the palmitoylation of Cys130 and Cys901 drives the translocation of NLRP3 to the dispersed Golgi (dTGN), while the palmitoylation mediated by DHHC5 (regulated by ABHD17A de-palmitoylation) and DHHC17-mediated palmitoylation of Cys419 enhance the initial binding of NLRP3 to NEK7’s LRR domain and the secondary binding of NACHT domain to NEK7, significantly strengthening the NLRP3-NEK7 interaction, thereby directly promoting the assembly of the inflammasome. In the termination stage, the palmitoylation catalyzed by DHHC12 (mediated by Cys844) promotes the binding of NLRP3 to the molecular chaperone HSC70, guiding its degradation through the molecular chaperone-mediated autophagy (CMA) pathway, and the palmitoylation mediated by PPT1 (regulated by Cys8 de-palmitoylation) also reduces the stability of NLRP3. Both of these processes jointly negatively regulate the duration of the inflammatory signal. Therefore, the different sites of palmitoylation precisely coordinate the subcellular localization, protein interaction, complex assembly, and protein stability of NLRP3 through spatiotemporal specificity and enzyme specificity mechanisms, ultimately achieving precise control over the activation of the inflammasome and the intensity of the inflammatory response (133). Additionally, ZDHHC17 mediates the palmitoylation of NLRP3 at the Cys419 residue. ZDHHC17 interacts with NLRP3 and facilitates its binding with NIMA-related kinase 7 (NEK7), thereby enhancing NLRP3 activity. The palmitoylation inhibitor 2-bromopalmitate can block NLRP3 palmitoylation and effectively inhibit NLRP3 activation *in vitro*. In a mouse colitis model, treatment with 2-bromopalmitate alleviated weight loss, improved survival, and ameliorated colonic pathological changes (134).

Epithelial ovarian cancer (EOC) utilizes the tricarboxylic acid (TCA) cycle and oxidative phosphorylation to sustain anabolic metabolism. In high-grade serous ovarian cancer (HGSOC) patient samples, ZDHHC18-mediated palmitoylation of malate dehydrogenase 2 (MDH2) at Cys138 enhances its enzymatic activity. This modification sustains mitochondrial respiration and accelerates malignant progression. Silencing MDH2 suppresses mitochondrial respiration and ovarian cancer cell proliferation, indicating that targeting ZDHHC18-mediated MDH2 palmitoylation represents a potential therapeutic strategy for EOC (135). In pancreatic ductal adenocarcinoma (PDAC), KRAS signaling upregulates ZDHHC20 expression in KPC mouse models, with its overexpression correlating with poor patient prognosis. ZDHHC20 palmitoylates YTHDF3 at Cys474, inhibiting its autophagic degradation. This leads to MYC accumulation and promotes pancreatic cancer progression. YTHDF3-derived peptide inhibitors competitively block ZDHHC20-mediated palmitoylation, downregulating MYC expression and inhibiting KRAS-mutant PDAC progression (**Figure 1F**) (136). Furthermore, *in vivo* shRNA screening identifies ZDHHC20 as essential for PDAC metastatic growth, though it does not affect *in vitro* proliferation or primary tumor development. This pro-metastatic function depends on tumor cell interactions with innate immunity, as evidenced by abolished effects in immunocompromised and NK-depleted models. Chemical genetic approaches have identified specific ZDHHC20 substrates that drive PDAC metastasis (137). Notably, DHHC20 inhibition sensitizes KRAS/EGFR-mutant cells—but not KRAS-wild-type cells—to the EGFR inhibitor gefitinib. This occurs through loss of EGFR palmitoylation at C-terminal cysteines. The palmitoylation-deficient mutant EGFRC1025A requires activated KRAS to confer gefitinib sensitivity. Moreover, DHHC20 inhibition overcomes resistance in EGFRT790M-mutant lung cancer cells. Combined treatment with 2-bromopalmitate and gefitinib induces cell death in gefitinib-resistant lines like NCI-H1975 (138).

### 3.2 Tumor-suppressive ZDHHCs ​

The hypermethylated gene ZDHHC1 inhibits tumor growth upon restored expression. Its substrate p53 undergoes palmitoylation at Cys135, Cys176, and Cys275—a novel modification essential for p53 nuclear translocation and tumor suppressor function. Notably, p53 recruits DNMT3A to the ZDHHC1 promoter, inducing hypermethylation and establishing an epigenetic feedback loop that inactivates p53 independently of genetic mutations (139). DHHC2 catalyzes Nrf2 palmitoylation, stabilizing its nuclear localization by inhibiting ubiquitination and delaying proteasomal degradation (**Figure 1G**). Inhibiting Nrf2 palmitoylation via 2-bromopalmitate (2-BP) or DHHC2 knockdown enhances Nrf2’s tumor-suppressive effects in gastric cancer (140). Separately, 2-BP inhibits colorectal cancer growth by targeting β-catenin palmitoylation (141). DHHC2 also palmitoylates cytoskeleton-associated protein 4 (CKAP4), enabling its ER-to-plasma membrane trafficking and nuclear localization (**Figure 1G**). CKAP4—a receptor for the antiproliferative factor (APF)—requires DHHC2-mediated palmitoylation to regulate E-cadherin, filamin, and ZO-1 expression. DHHC2 knockdown disrupts APF-mediated suppression of tumor proliferation, confirming DHHC2’s tumor suppressor role (142). zDHHC4 palmitoylates pericyte-expressed KAI1, enabling membrane localization. KAI1 induces LIF release via Src/p53 signaling and directly binds VEGF/PDGF to inhibit angiogenesis. *In vivo*, KAI1 supplementation suppresses tumor angiogenesis and growth (143). DHHC7 and DHHC21 palmitoylate steroid receptors (ER, PR, AR), enabling membrane localization and signaling in cancer cells. Their knockdown disrupts receptor function, highlighting their potential as therapeutic targets (144). In diffuse large B-cell lymphoma (DLBCL), ZDHHC21 acts as a tumor suppressor by palmitoylating FASN at Cys1317, reducing FASN stability and fatty acid synthesis. ZDHHC21 downregulation correlates with poor prognosis. Mahuangoside C (FDA-approved) stabilizes ZDHHC21 and suppresses DLBCL growth (**Figure 1H**) (145). In prostate cancer, ZDHHC7 downregulation correlates with poor outcomes. ZDHHC7 suppresses androgen receptor (AR) transcription, inhibiting proliferation and invasion. ZDHHC7 restoration reverses oncogenic phenotypes *in vitro* and *in vivo* (146). In non-tumor diseases, there are also studies reporting that DHHC7 is involved in the process of pyroptosis. Pyroptosis is a destructive and programmed form of cell death mediated by pyroptosis proteins (GSDMs), among which GSDMD plays a significant role in innate immunity and pathological processes (147). The palmitoylation mediated by DHHC7 is necessary for the cleavage of GSDMD by caspase and the activation of its active fragment GSDMD-NT, which targets the cell membrane. Subsequently, the de-palmitoylation mediated by APT2 is a key step in the oligomerization of GSDMD-NT on the membrane to form pores. This “palmitoylation-de-palmitoylation relay” mechanism precisely controls the temporal and spatial sequence of the activation of the pyroptosis execution protein GSDMD, and is crucial for the body’s resistance to infection and regulation of inflammatory responses. Disrupting this regulatory pathway will significantly affect the occurrence of pyroptosis and the outcome of infectious diseases in the body (44).

#### 3.2.1 Depalmitoylation

**Figure 2:**
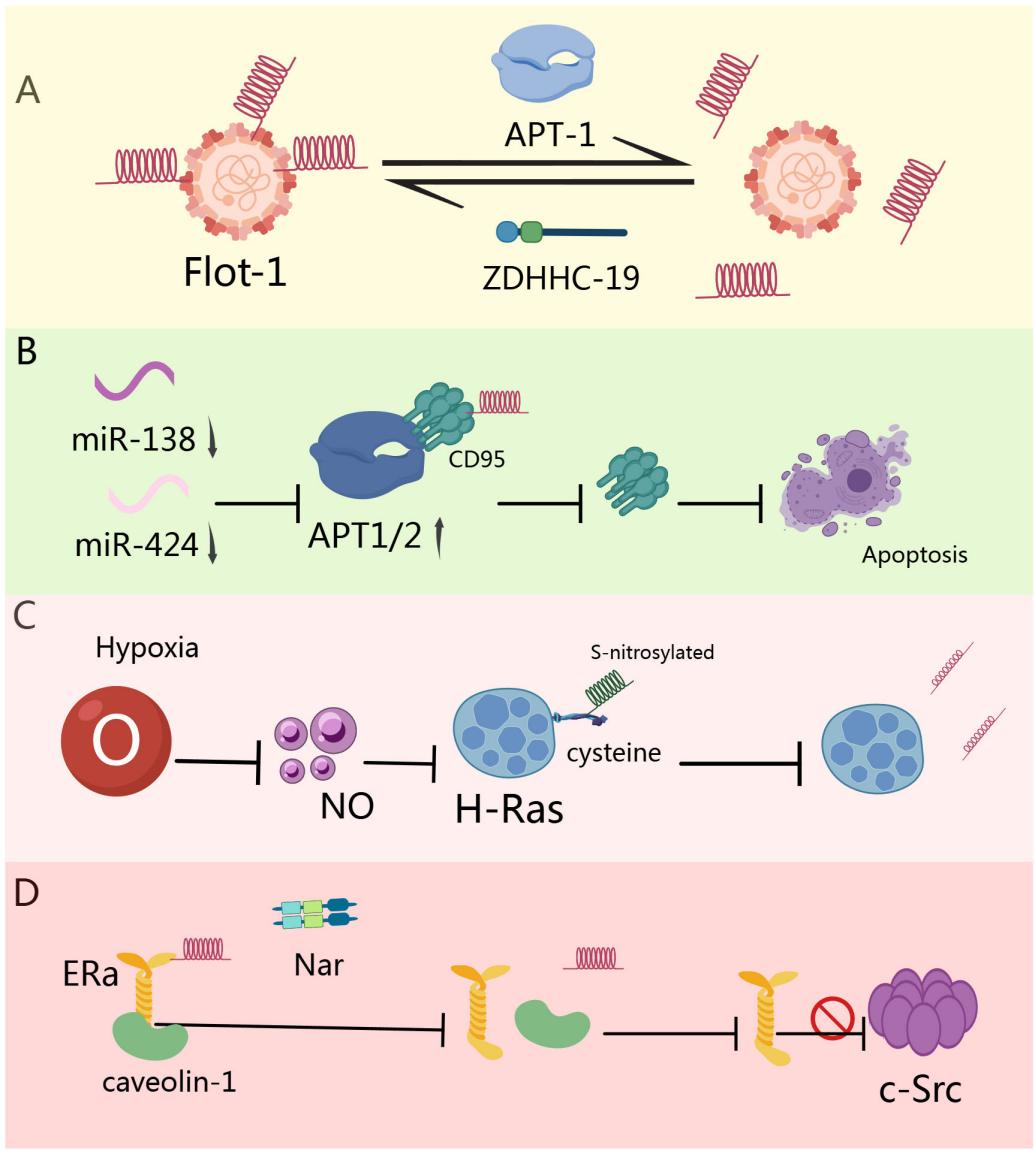
Role of depalmitoylation in tumors. **(A)** miR-138 and miR-424 are downregulated in CLL cells, leading to the overexpression of APT1 and 2. APT directly interacts with CD95, promoting its depalmitoylation, thereby inhibiting CD95-mediated apoptosis (149). **(B)** High concentrations of hypoxia induce nitric oxide production, resulting in the S-nitrosylation of terminal cysteine in H-Ras, which triggers H-Ras depalmitoylation and mislocalization (150). **(C)** Nar rapidly depalmitoylates ERα, leading to its dissociation from caveolin-1, thereby hindering the binding of ERα with adaptor proteins and signaling proteins (c-Src) involved in the mitogenic signaling cascade (151). **(D)** APT-1 catalyzes the depalmitoylation of Flot-1, while ZDHHC-19 catalyzes the repalmitoylation of Flot-1 after depalmitoylation, thereby promoting the transformation of cervical cancer cells (148).

Insulin-like growth factor-1 (IGF-1) signaling through IGF-1 receptor (IGF-1R) induces palmitoylation turnover of Flotillin-1 (Flot-1) at the plasma membrane, promoting cell proliferation. Mechanistically, acyl protein thioesterase-1 (APT-1) catalyzes Flot-1 depalmitoylation while zDHHC19 mediates its repalmitoylation—a cycle facilitating cervical cancer transformation. This dynamic modification prevents IGF-1R endocytosis and lysosomal degradation, causing receptor overactivation. In malignant cervical tissues, elevated FLOT1, LYPLA1, and ZDHHC19 cooperatively upregulate TIAM1 and GREM1 to induce epithelial-mesenchymal transition (EMT). Blocking this palmitoylation cycle inhibits EMT, migration, and invasion (**Figure 2A**) (148). In chronic lymphocytic leukemia (CLL), downregulated miR-138 and miR-424 cause APT1/2 overexpression. These key depalmitoylases significantly reduce membrane protein palmitoylation in CLL. APT1/2 directly interact with CD95, promoting its depalmitoylation and inhibiting CD95-mediated apoptosis. Restoring apoptosis through APT inhibition, miR-138/-424 supplementation, or pharmacological intervention highlights APT’s critical role in regulating CD95 death signaling (**Figure 2B**) (149). Hypoxia induces nitric oxide production, triggering S-nitrosylation of H-Ras at its C-terminal cysteine. This modification promotes H-Ras depalmitoylation and mislocalization. In PC12 cells, hypoxia/nitric oxide significantly reduces H-Ras palmitoylation, altering ERK phosphorylation and metabolic pathways—revealing new therapeutic opportunities (**Figure 2C**) (150). Naringenin (Nar), a bioactive flavonoid, exerts anticancer effects through dual estrogenic/anti-estrogenic activities. Nar induces rapid ERα depalmitoylation, dissociating it from caveolin-1 and disrupting interactions with c-Src signaling complexes. Concurrently, Nar activates p38 kinase via palmitoylation-independent ER mechanisms, collectively inhibiting cancer proliferation (**Figure 2D**) (151).

## 4 Palmitoylation and tumor signaling pathways

### 4.1 Hippo-associated signaling pathways

**Figure 3:**
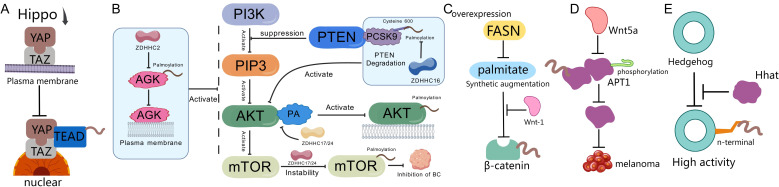
Palmitoylation and tumor signaling pathways. **(A)** When the Hippo pathway is inactivated, YAP/TAZ proteins translocate from the cytoplasm to the nucleus, where they bind to TEAD transcription factors, promoting the transcription of pro-growth and anti-apoptotic genes, thereby enhancing cancer cell proliferation, metastasis, and therapeutic resistance (154). **(B)** PA can promote the palmitoylation and activation of AKT via ZDHHC17/24, anchoring it to the cell membrane (160). After being palmitoylated at Cysteine 600 by ZDHHC16, PCSK9 shows increased affinity for binding to PTEN, inducing PTEN degradation and activating AKT (19). ZDHHC22 reduces mTOR stability through palmitoylation, decreasing the activation of the AKT signaling pathway, thereby inhibiting breast cancer cell proliferation both *in vitro* and *in vivo* (167). ZDHHC2 promotes AGK translocation to the plasma membrane and activates the PI3K-AKT-mTOR signaling pathway by mediating AGK S-palmitoylation, thereby modulating sensitivity to sunitinib (162). **(C)** Overexpression of FASN leads to increased synthesis of palmitate, which stabilizes the accumulation and activation of β-catenin in the cytoplasm through Wnt-1-mediated palmitoylation (170). **(D)** Wnt5a signaling increases melanoma invasiveness by promoting APT1-mediated depalmitoylation of pro-metastatic cell adhesion molecules CD44 and MCAM (172). **(E)** The N-terminal of Hedgehog protein requires palmitoylation by the MBOAT family multi-transmembrane enzyme Hedgehog acyltransferase (Hhat) to achieve high activity (179).

The Hippo pathway critically regulates tissue growth, organ size, and cancer suppression. Its dysregulation drives uncontrolled proliferation and tumorigenesis, while also governing stem cell maintenance, regeneration, and development. Central to this pathway are TEAD transcription factors and their co-activators YAP/TAZ, which orchestrate gene expression programs essential for cell growth, differentiation, and organ size control. TEAD palmitoylation is indispensable for protein stability and activity, modulating core domain hydrophobicity through lipid tail extensions. Biochemical and structural studies reveal that palmitoylation occurs within conserved hydrophobic cavities in TEAD2 and TEAD3, ensuring proper protein folding and stability (153). This modification regulates TEAD protein levels and transcriptional activity, with dysregulation severely impairing function (152). When Hippo signaling is inactivated, nuclear-translocated YAP/TAZ bind palmitoylated TEAD to drive pro-growth and anti-apoptotic gene expression, promoting cancer proliferation, metastasis, and therapy resistance. Critically, TEAD palmitoylation facilitates YAP/TAZ-TEAD interactions (**Figure 3A**) (154). Recent studies identified JM7—a novel small-molecule inhibitor that binds TEAD’s lipid pocket, disrupts palmitoylation, and induces destabilization. JM7 suppresses YAP target gene expression, inhibits cancer cell proliferation, colony formation, and migration across multiple lines, positioning it as a promising therapeutic lead (155). Loss of TEAD palmitoylation also prevents chromatin binding, inactivating downstream Hippo target genes. Virtual screening identified potent TEAD2 palmitoylation inhibitors (ChEBML196567, ZINC000013942794) with superior binding affinity and drug-like properties (156).

### 4.2 Palmitoylation-mediated cross-pathway regulation in cancer

Palmitoylation serves as a critical nexus for oncogenic signaling crosstalk, particularly between EGFR and PI3K-AKT pathways. In non-small cell lung cancer (NSCLC), EGFR palmitoylation regulates the interaction between PI3K regulatory subunit PIK3R1 (p85) and EGFR, enhancing recruitment of PI3K heterodimers to the plasma membrane and amplifying downstream AKT activation. Knocking down palmitoyltransferase DHHC20 or expressing palmitoylation-resistant EGFR mutants reduces PI3K/MYC signaling and cell proliferation (157). Therapeutically, targeting this axis improves EGFR tyrosine kinase inhibitor (TKI) sensitivity: DHHC20 inhibition increases cancer cell dependence on EGFR signaling, sensitizing tumors to TKIs (158). This cross-pathway crosstalk drives treatment resistance. In TKI-resistant NSCLC, palmitoylation sustains kinase-inactive EGFR dimerization, maintaining persistent signaling. Disrupting palmitoylation (via cysteine mutations or DHHC20 inhibition) eliminates aberrant dimerization and overcomes resistance (159). Similarly, in hepatocellular carcinoma (HCC), PCSK9 palmitoylation at Cys600 by ZDHHC16 enhances PTEN degradation, activating AKT-S473 phosphorylation and conferring sorafenib resistance (19). Clinically relevant solutions: PCSK9-derived peptides competitively inhibit palmitoylation, suppress AKT activation, and restore sorafenib efficacy (19). Beyond EGFR crosstalk, palmitoylation directly regulates AKT activation. High-fat-diet-induced HCC promotes palmitic acid (PA)-driven AKT palmitoylation via ZDHHC17/24, anchoring AKT to membranes in a PIP3-independent manner and preventing inactive polymerization (160). Therapeutic interventions: Orlistat (FASN inhibitor) limits PA synthesis, reducing AKT palmitoylation and suppressing liver tumors (160, 161). In clear cell renal cell carcinoma (ccRCC), ZDHHC2-mediated S-palmitoylation of AGK activates PI3K-AKT-mTOR signaling, driving sunitinib resistance (162). Palmitoylation critically modulates EGFR stability and trafficking. In colorectal cancer, lipid-rich microenvironments upregulate FASN, enhancing EGFR palmitoylation and plasma membrane stabilization (163). Conversely, CD82 palmitoylation at Cys5/Cys74 promotes EGFR internalization in breast cancer (164). Notably, Orlistat inhibits FASN, inducing EGFR ubiquitination and eliminating oncogenic signaling in NSCLC (161). Cross-pathway integration extends to transcriptional regulation. In EGFR-activated cancers, AKT phosphorylates TSPAN8 (Ser129), enabling its palmitoylation/cholesterol-dependent nuclear translocation with 14-3-3θ/importin-β. Nuclear TSPAN8 stabilizes STAT3 chromatin binding, upregulating MYC/BCL2/MMP9 and driving aggressiveness (165). Therapeutic relevance: Targeting the EGFR-AKT-TSPAN8-STAT3 axis may benefit refractory cancers. Combination therapies show synergy: Co-inhibition of TEAD palmitoylation and AKT induces cancer cell death (166). Palmitoyltransferase inhibitors (e.g., against ZDHHC16 or DHHC20) sensitize tumors to targeted therapies (19, 158). Metabolic interventions (Orlistat, FASN blockers) disrupt palmitate supply for oncogenic palmitoylation (160, 161). Palmitoylation-resistant mutants provide templates for peptide-based therapeutics (19, 158). Furthermore, there are studies indicating that in breast cancer cases, ZDHHC22 palmitoylates mTOR, suppressing AKT signaling and restoring tamoxifen sensitivity (167). Prostate cancer progression involves Cav-1 palmitoylation, which modulates Src/AKT/EGFR interactions (168). DKK1-induced CKAP4/LRP6 de-palmitoylation relocates them to non-DRM membranes via PI3K-AKT activation (169). We summarize palmitoylation-mediated crosstalk in the PI3K/PTEN/AKT/mTOR pathway in **Figure 3B**.

### 4.3 Wnt-associated signaling pathways

In prostate cancer, FASN overexpression increases palmitate ester synthesis and stabilizes β-catenin accumulation through Wnt-1-mediated palmitoylation, driving oncogenesis (**Figure 3C**) (170). The novel PORCN inhibitor WHN-88—featuring a unique diiodopyridinone structure—effectively blocks Wnt ligand palmitoylation, inhibiting their secretion and downstream signaling to provide a therapeutic strategy for Wnt-driven cancers (171). In melanoma, Wnt5a signaling phosphorylates APT1, enhancing its depalmitoylation activity while reducing dimerization. This APT1 phosphorylation promotes melanoma invasion *in vitro* and correlates with advanced tumor grade and metastasis, suggesting APT1 inhibition as a therapeutic strategy for Wnt5a-driven cancers (**Figure 3D**) (172). Wnt5a further regulates cell polarity by depalmitoylating melanoma cell adhesion molecule (MCAM) at Cys590. Mutation of Cys590 to glycine mimics Wnt5a-induced MCAM polarity. APT1 inhibition blocks Wnt5a-mediated MCAM depalmitoylation, asymmetric localization, and invasion. Direct manipulation of basal palmitoylation mechanisms enhances invasion, while cancer-associated palmitoyltransferase mutations reduce MCAM palmitoylation and weaken its invasion-suppressing function. These findings establish Wnt5a-induced depalmitoylation as critical for protein polarity and invasion (173). APT1-mediated depalmitoylation also maintains dynamics of Notch/Wnt proteins, gene expression, and asymmetric cell division (174).

Porcupine (PORCN), a membrane-bound O-acyltransferase, is essential for Wnt palmitoylation, secretion, and bioactivity. Studies evaluating the PORCN inhibitor Wnt-C59 (C59) demonstrate its nanomolar *in vitro* efficacy and oral bioavailability in mice. C59 inhibits mammary tumor development in MMTV-WNT1 transgenic mice by downregulating Wnt/β-catenin targets without significant toxicity (175). Wnt signaling inhibitors (IWPs) antagonize Wnt pathways by blocking PORCN-mediated palmitoylation. IWPs are ATP-competitive inhibitors of wild-type CK1δ and M82FCK1δ, with IWP-2 showing specificity among 320 kinases and broad anticancer activity. Improved IWP-derived CK1 inhibitors suggest effects beyond PORCN to CK1δ/ϵ pathways (175). In colorectal cancer, Wnt secretion requires PORCN palmitoylation. PORCN inhibitor IWP2 blocks epithelial transition in mesenchymal LIM1863-Mph cells, upregulating Wnt genes—particularly Wnt2B. Recombinant Wnt2B overcomes IWP2 inhibition by collaborating with Frizzled7 to mediate mesenchymal-epithelial transition (MET) (176). Scaffold hybridization strategies yielded lead compound 62, exhibiting sub-nanomolar Wnt inhibition (IC50 = 0.11 nM) in reporter assays. Compound 62 suppresses Wnt protein secretion, confirming direct PORCN targeting, while demonstrating favorable chemical and metabolic stability—supporting further development of potent Wnt inhibitors (177).

### 4.4 Hedgehog-associated signaling pathways

Research demonstrates that Hedgehog family protein overexpression critically drives oncogenesis in multiple cancers. Functional Sonic Hedgehog (Shh) signaling requires N-terminal palmitoylation catalyzed by Hedgehog acyltransferase (Hhat). To precisely quantify this process, a novel Microfluidic Mobility Shift Assay (MSA) was developed. MSA enables real-time quantitative analysis of palmitoylated Shh, facilitating studies of Hhat catalytic mechanisms, kinetics, and small-molecule inhibitor efficacy. This technique overcomes limitations of traditional methods by providing direct measurement of lipid modifications (**Figure 3E**) (178).

Hedgehog proteins require N-terminal palmitoylation by the MBOAT-family multipass transmembrane enzyme Hhat for full activity. In PDAC cell line PANC-1 and transfected HEK293a cells, Hhat localizes to the endoplasmic reticulum (ER). Hhat is essential for Shh palmitoylation, formation of high-molecular-weight extracellular complexes, and functional activity. Hhat knockout inhibits autocrine/juxtacrine Hh signaling and suppresses PDAC cell growth and invasion *in vitro*. In Shh-expressing HEK293a and A549 NSCLC cells, Hhat knockdown impairs juxtacrine/paracrine signaling to C3H10T1/2 and Shh-Light2 reporter cells, underscoring Hhat’s critical role in Hh-dependent tumorigenesis (179).

Hedgehog protein maturation involves N-terminal palmitate addition and C-terminal cholesterol modification—both essential for function and localization. Structural studies reveal HHAT possesses 10 transmembrane domains with two reentrant loops, positioning key His/Asp residues across the ER membrane. HHAT undergoes palmitoylation at multiple cytoplasmic cysteines, crucial for its membrane stability. Critically, mutations in conserved catalytic-domain His residues abolish HHAT’s ability to palmitoylate Hedgehog proteins, revealing new insights into its intracellular mechanism (180).

### 4.5 Others

Beyond classical pathways, palmitoylation regulates additional oncogenic signaling cascades.

The small GTPase RAB27B activates NRAS signaling by facilitating NRAS palmitoylation and membrane trafficking. RAB27B overexpression in myeloid malignancies with CBL or JAK2 mutations correlates with poor acute myeloid leukemia (AML) prognosis. RAB27B loss inhibits growth in CBL-deficient or NRAS-mutant cell lines. *In vivo*, Rab27b deletion abrogates mutation-driven progenitor expansion, ERK signaling, and NRAS palmitoylation, suppressing myelomonocytic leukemogenesis. Mechanistically, RAB27B governs NRAS palmitoylation via ZDHHC9 interaction, thereby modulating c-RAF/MEK/ERK signaling to impact leukemia progression. Critically, RAB27B depletion suppresses oncogenic NRAS signaling and leukemic growth in primary human AML, while RAB27B expression predicts MEK inhibitor sensitivity (181).

Baicalin modulates PLSCR1 and N-RAS palmitoylation in primary AML cells, promoting their nuclear translocation or Golgi trafficking. These alterations inactivate the N-RAS/RAF1 pathway (182). In hypopharyngeal squamous cell carcinoma (HPSCC), elevated DHHC9/DHHC15 expression drives tumorigenesis. The palmitoylation inhibitor 2-bromopalmitate (2BP) reduces proliferation, invasion, and migration without inducing apoptosis. 2BP decreases Ras palmitoylation, membrane localization, and FGF/ERK signaling (183).

In hepatocellular carcinoma (HCC), ZDHHC7 overexpression correlates with poor outcomes. DHHC7 mediates reversible STAT3 palmitoylation at Cys108, enhancing its transcriptional activity. Palmitoylated STAT3 upregulates HIF1A, increasing HIF1α protein. DHHC7 inhibition reduces STAT3 palmitoylation and HIF1α abundance. Cyclin-dependent kinase 5 (CDK5) stabilizes HIF1α, promoting ZDHHC7 expression and forming a DHHC7-STAT3-HIF1α positive feedback loop. Disrupting this axis suppresses HCC growth *in vivo* (184).

In glioblastoma stem cells (GSCs), local anesthetics suppress IL-6/STAT3 signaling by reducing ZDHHC15 transcription, GP130 palmitoylation, and membrane localization (52). In neuropathic cancer pain (NCP), spinal dorsal horn astrocyte activation progresses with pain severity, upregulating palmitoyltransferase ZDHHC23 and GFAP palmitoylation. This enhances secretion of CXCL-10, IL-6, and GM-CSF, further activating astrocytes via STAT3 signaling (185).

## 5 Palmitoylation and anti-tumor immunity

In the tumor microenvironment (TME), metabolic reprogramming of tumor-associated macrophages (TAMs) serves as a key driver of immunosuppression. Studies demonstrate that in hepatocellular carcinoma (HCC), TAMs regulate the activity of serine palmitoyltransferase (SPT) via the kinase NEK2, thereby promoting the biosynthesis of sphingosine-1-phosphate (S1P). S1P acts as an immunosuppressive factor derived from TAMs, accelerating tumor progression and conferring resistance to immunotherapy by promoting the expansion of regulatory T cells (Tregs) while suppressing effector T cell function. Targeting NEK2 or S1P reverses the immunosuppressive phenotype of TAMs and enhances the efficacy of immune checkpoint blockade therapy (186). Furthermore, palmitoylated lipopeptides (e.g., the di-palmitoylated peptide Pam2IDG) promote dendritic cell maturation through activation of TLR2/6 signaling; however, their antitumor efficacy is hampered by TAM-mediated suppression. Depletion of TAMs or blockade of IL-10 and COX-2 significantly augments the immunotherapeutic effect of palmitoylated lipopeptides (187). The accumulation of myeloid-derived suppressor cells (MDSCs) represents a critical mechanism for tumor immune evasion. In acute myeloid leukemia (AML), tumor-derived extracellular vesicles (EVs) activate TLR2 signaling on monocytes via their surface palmitoylated proteins, inducing their differentiation into immunosuppressive CD14^+^HLA-DR^low^ MDSCs. This process is dependent on Akt/mTOR pathway activation and glycolytic metabolic reprogramming, accompanied by upregulation of immunosuppressive factors such as IDO and S100A8/9. Targeting protein palmitoylation in AML cells blocks EV-mediated MDSC differentiation and restores antitumor immune responses (188). In pancreatic ductal adenocarcinoma (PDAC), the palmitoyltransferase ZDHHC20 promotes metastatic outgrowth by palmitoylating unidentified substrate(s), an effect dependent on NK cell-mediated immune evasion mechanisms (137). Additionally, chemotherapeutic agents (e.g., cisplatin) induce tumor release of oxidized phospholipids (oxPAPC), which recruit MDSCs via palmitoylation-dependent MCP-1/CCL2 and LTB4/LTB4R pathways, ultimately leading to chemoresistance (189). Palmitoylation directly suppresses NK cell function by regulating immune checkpoint molecules and metabolites. TIM-3, a key inhibitory receptor on NK cells, undergoes palmitoylation mediated by DHHC9 at cysteine residue 296 (Cys296). This modification prevents binding of the E3 ubiquitin ligase HRD1, thereby inhibiting TIM-3 ubiquitination and degradation, consequently promoting NK cell exhaustion. Targeting DHHC9 or employing palmitoylation inhibitors accelerates TIM-3 degradation and restores the antitumor activity of NK cells (190). In breast cancer, ablation of the palmitoyltransferase DHHC3 enhances NK cell-mediated tumor clearance by inducing tumor cell oxidative stress and senescence (119). Furthermore, the accumulation of long-chain acylcarnitines (e.g., palmitoyl-carnitine) in the HCC microenvironment indirectly impairs NK cell immunosurveillance by inducing iNKT cell senescence and diminishing their cytotoxicity (191). Clinical translation strategies targeting these mechanisms have been proposed. PPT1 inhibitors (e.g., GNS561) activate the cGAS-STING pathway to induce type I interferon secretion, promote macrophage polarization towards the M1 phenotype, reduce MDSC infiltration, and enhance the efficacy of anti-PD-1 therapy (192, 193). DHHC3/DHHC9 inhibitors hold potential for restoring T cell and NK cell function, warranting exploration in clinical trials combined with immune checkpoint blockade (119, 190). Targeting lipid metabolism (e.g., clearance of palmitoyl-carnitine) or combining with IL-2 may reverse iNKT cell senescence and augment NK cell activity (191, 194).

**Figure 4:**
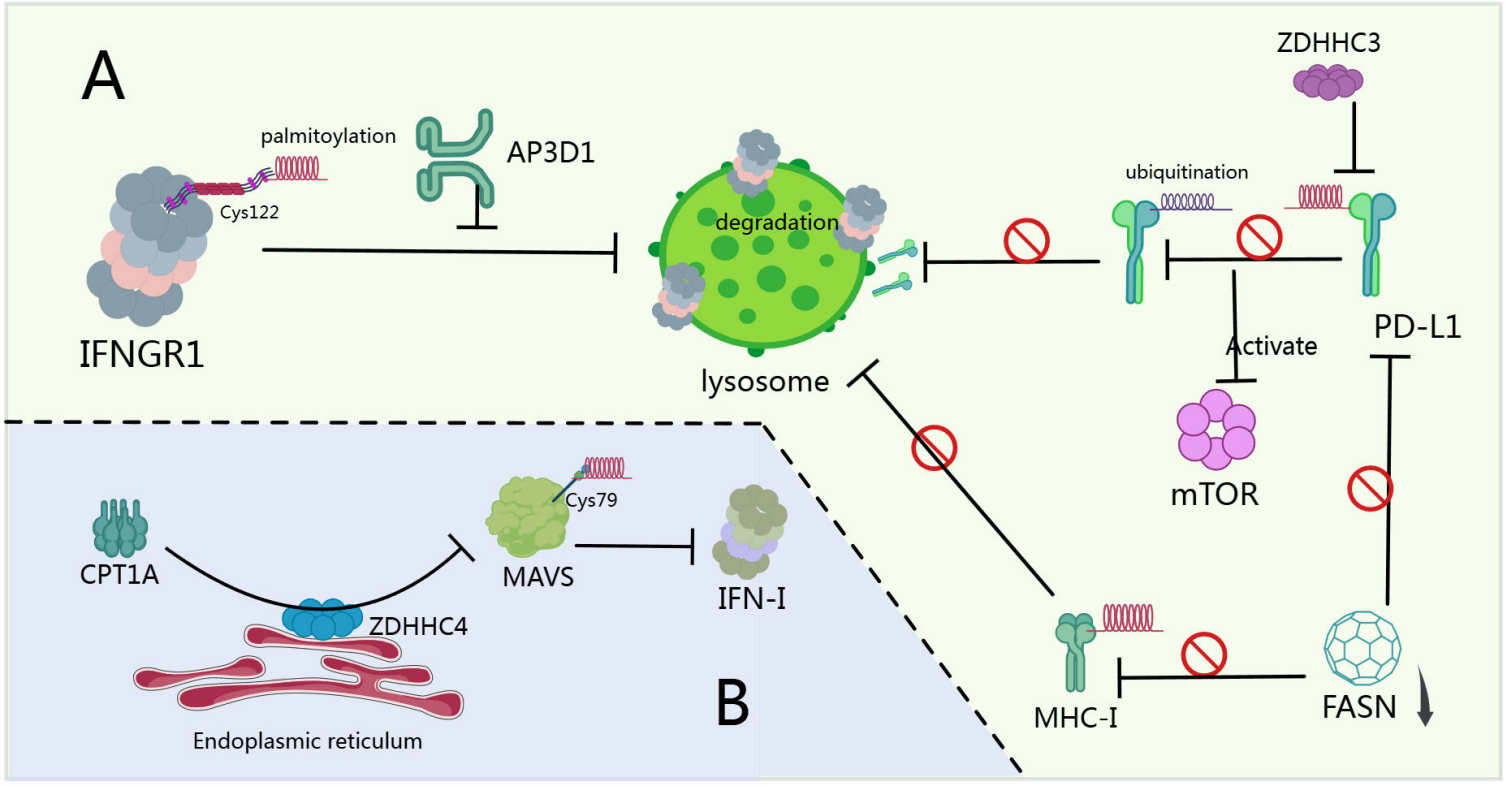
Palmitoylation and anti-tumor immunity. **(A)** After IFNGR1 is S-palmitoylated at Cys122, it is sorted by AP3D1 to the lysosome for degradation (23). ZDHHC3 mediates the palmitoylation of PD-L1 in its cytoplasmic domain, and this modification stabilizes PD-L1 by blocking its ubiquitination, thereby preventing lysosomal degradation. Palmitoylation of PD-1 promotes the activation of the mTOR signaling pathway and tumor cell proliferation (17, 195). FASN inhibition reduces MHC-I palmitoylation, preventing its lysosomal degradation (197). Pharmacological inhibition of FASN effectively suppresses PD-L1 palmitoylation and expression (198). **(B)** CPT1A recruits endoplasmic reticulum-localized ZDHHC4 to catalyze the palmitoylation of MAVS at Cys79, thereby stabilizing and activating MAVS. Enhanced palmitoylation of MAVS amplifies the IFN-I response (196).

Palmitoylation critically regulates anti-tumor immunity and tumor progression. Programmed Death-Ligand 1 (PD-L1) undergoes intracellular storage and membrane redistribution, diminishing checkpoint blockade efficacy. Cytoplasmic domain palmitoylation stabilizes PD-L1 by blocking ubiquitination and lysosomal degradation (**Figure 4A**). ZDHHC3 is the primary palmitoyltransferase for PD-L1 modification. Inhibiting PD-L1 palmitoylation via 2-bromopalmitate or DHHC3 silencing activates anti-tumor immunity *in vitro* and *in vivo*. PD-L1 palmitoylation competitive inhibitors reduce tumor PD-L1 expression and enhance T-cell immunity (17). Parallelly, specific DHHC enzymes palmitoylate PD-1, enhancing its stability by preventing lysosomal degradation. This activates mTOR signaling and promotes tumor proliferation (**Figure 4A**) (195). Targeting epigenetic regulators enhances anti-PD-1 immunotherapy by activating Type I interferon (IFN-I) responses. CPT1A recruits ER-localized ZDHHC4 to palmitoylate MAVS at Cys79, stabilizing it through altered ubiquitination (inhibiting K48-linked, promoting K63-linked). Increased CPT1A amplifies MAVS palmitoylation and IFN-I responses, improving viral control and anti-tumor immunity. CPT1A inducers enhance epigenetic therapy combined with PD-1 blockade in refractory tumors (**Figure 4B**) (196). In HCC, FASN inhibition reduces MHC-I palmitoylation, preventing lysosomal degradation and increasing MHC-I expression (**Figure 4A**). This enhances antigen presentation and CD8+ T-cell activation. DHHC3 directly binds and negatively regulates MHC-I. FASN deficiency promotes CD8+ T-cell infiltration and tumor killing. Combining FASN inhibitors (orlistat/TVB-2640) with anti-PD-L1 antibodies suppresses tumor growth (197). In chemoresistant bladder cancer, FASN inhibition suppresses palmitoylated PD-L1 expression, suggesting targeted therapy potential (**Figure 4A**) (198). Additionally, nanoparticle-based PD-L1 inhibitors (FRS) with fluoralkylated competitive peptides disrupt endogenous PD-L1. FRS combined with doxorubicin reduces PD-L1 abundance and induces immunogenic cell death in colon cancer models (199). In pancreatic cancer, ZDHHC9 overexpression correlates with impaired immunity. ZDHHC9 knockdown converts “cold” to “hot” tumor microenvironments, inhibiting progression and extending survival. ZDHHC9 deficiency sensitizes tumors to anti-PD-L1 therapy via CD8+ T-cell involvement (200). Colorectal cancers resistant to immunotherapy show disrupted IFN/MHC signaling. Optineurin maintains pathway integrity by blocking IFNGR1 palmitoylation (Cys122)-dependent lysosomal sorting via AP3D1. Targeting IFNGR1 palmitoylation stabilizes IFNGR1, enhances tumor immunity, and sensitizes to checkpoint therapy (**Figure 4A**) (23). In AML, lipid transporter CD36 promotes immune evasion. CD36 senses OxLDL, initiating TLR4-LYN-MYD88-NF-κB signaling and enhancing CD36 palmitoylation via exogenous palmitate transfer. This activates MYD88-mediated pathways, and NF-κB induces immunosuppressive genes. High-fat diets or decitabine treatment amplify CD36-mediated immune suppression. Statins enhance decitabine efficacy by countering CD36-driven immunosuppression (201).

Palmitoylation, as a dynamic and reversible post-translational modification, directly drives immunotherapy resistance across diverse cancers by regulating the stability, subcellular localization, and functional activity of key immune signaling proteins. Previous studies indicate that palmitoylation can disrupt the integrity of antitumor immune signaling pathways to modulate therapeutic efficacy. In colorectal cancer, optineurin deficiency promotes AP3D1-mediated lysosomal sorting and degradation of S-palmitoylated IFNGR1 (at Cys122), thereby blocking IFNγ signal transduction. Degradation of IFNGR1 directly suppresses MHC-I expression and impairs CD8^+^ T cell recognition capacity, ultimately leading to immune evasion. Pharmacological inhibition of IFNGR1 palmitoylation stabilizes IFNGR1, restores T cell function, and reverses treatment resistance (23). Additionally, acute myeloid leukemia (AML) cells uptake exogenous palmitate via CD36, activating ZDHHC6-mediated MYD88 palmitoylation to potentiate the TLR4-LYN-MYD88-NF-κB signaling axis. NF-κB subsequently drives the expression of immunosuppressive genes (e.g., IL-10, TGF-β), directly inhibiting CD8^+^ T cell activity. High-fat diet or decitabine treatment exacerbates AML immunosuppression through this mechanism, while statins can reverse resistance by blocking CD36-mediated signaling (201). Evidence further demonstrates that palmitoylation maintains the stability of immune checkpoint proteins. In tumor cells, DHHC3 (ZDHHC3) catalyzes PD-L1 palmitoylation to facilitate its plasma membrane localization and prevent lysosomal degradation. The chimera degrader cp-PCCs (e.g., PCC16) targeting DHHC3 effectively reduces PD-L1 levels and significantly suppresses tumor growth in immunotherapy-resistant models (202, 203). Disrupting PD-L1 palmitoylation with 2-bromopalmitate (2-BP) delivered via a chemotherapeutic co-loaded nanocarrier simultaneously eliminates cell-surface and exosomal PD-L1, overcoming therapeutic resistance (203). Moreover, ZDHHC9 overexpression promotes an immunosuppressive ‘cold tumor’ microenvironment. Its ablation enhances CD8^+^ T cell infiltration to establish ‘hot’ tumors, significantly improving anti-PD-L1 efficacy. This strategy is validated by ZDHHC9-silencing siRNA nanoparticles (200). Palmitoylation also enhances tumor cell resistance to ferroptosis. ZDHHC8-catalyzed palmitoylation of GPX4 augments its enzymatic activity and stability, protecting tumor cells from ferroptosis. Disruption of this modification restores lipid peroxidation sensitivity and promotes CD8^+^ T cell-induced ferroptotic death (204). In lung cancer stem cells (CSCs), CPT1A forms a positive feedback loop by inhibiting c-Myc ubiquitination and degradation, which activates the NRF2/GPX4 antioxidant pathway and downregulates ACSL4 to reduce polyunsaturated fatty acid (PUFA) accumulation, thereby conferring ferroptosis resistance. Targeting CPT1A enhances antitumor efficacy of immune checkpoint blockade (205, 206). Palmitoylation contributes to metabolism-driven T cell exhaustion. In hepatocellular carcinoma (HCC), Riplet deficiency stabilizes fatty acid synthase (FASN), increasing secretion of palmitic acid (PA/C16:0). PA induces terminal CD8^+^ T cell exhaustion by enhancing STAT3 palmitoylation, driving anti-PD-1 resistance. FASN inhibitors reverse T cell exhaustion and overcome this resistance (207). Tumor-associated macrophages (TAMs) in HCC employ NEK2 to regulate palmitoylation-associated sphingolipid metabolism, promoting S1P production. S1P confers immunotherapy resistance by activating regulatory T cells (Tregs) and suppressing effector T cell function. Targeting the NEK2/S1P axis restores immune responses (186). High PPT1 expression correlates with poor prognosis in HCC. Its inhibitor DC661 enhances CD8^+^ T cell activity by inhibiting palmitoylation-dependent autophagy pathways, reversing resistance to both sorafenib and immunotherapy (208). In summary, palmitoylation drives immunotherapy resistance through multifaceted mechanisms: direct modification of immune signaling proteins, stabilization of immune checkpoints, enhancement of anti-ferroptotic defenses, and remodeling of the metabolic microenvironment. These mechanisms provide actionable pathways for rational therapeutic combinations. Disrupting the palmitoylation regulatory network represents a promising precision strategy to overcome immunotherapy resistance.

## 6 Clinical applications

This section examines the clinical translational potential and future research trajectories of targeting protein palmitoylation in oncology. Palmitoylation represents a promising therapeutic frontier due to its fundamental role in tumor pathogenesis and immune regulation. We synthesize current advances in palmitoyltransferase inhibitors and depalmitoylase modulators, emphasizing their capacity to potentiate treatment efficacy and circumvent therapeutic resistance. Furthermore, we delineate emerging directions—including novel small-molecule discovery, biomarker-driven patient stratification, and rational combination strategies—that may optimize clinical outcomes in precision cancer medicine.

Current research explores diverse palmitoylation-targeting inhibitors—including 2-bromopalmitate (2-BP) and TEAD inhibitors—demonstrating significant therapeutic potential. 2-BP irreversibly inhibits palmitoyltransferase activity across all DHHC proteins (209). Building on earlier discussions of 2-BP’s anticancer properties, recent work in triple-negative breast cancer (TNBC) developed AFT/2-BP@PLGA@MD: an advanced biomimetic nanoplatform integrating targeted therapy and immunotherapy. This system encapsulates the palmitoylation inhibitor 2-BP within poly(lactic-co-glycolic acid) (PLGA) nanoparticles coated with tumor-derived membranes (MD), simultaneously enhancing afatinib’s (AFT) therapeutic efficacy against TNBC cells while blocking PD-1/PD-L1 checkpoint signaling. *In vitro*, 2-BP potentiates AFT’s suppression of tumor cell proliferation and migration. In murine models, AFT/2-BP@PLGA@MD nanoparticles significantly inhibit 4T1 tumor growth/metastasis, extend survival, and activate antitumor immunity—offering new therapeutic avenues for refractory TNBC (210).

The Hippo pathway critically drives tumor growth. Genetic ablation of YAP/TAZ combined with novel TEAD palmitoylation inhibitors significantly blocks and reverses schwannoma progression *in vitro* and *in vivo* (211). Building on this, SWTX-143—a covalent YAP/TAZ-TEAD inhibitor—irreversibly binds the palmitoylation pocket across all four TEAD isoforms, specifically suppressing YAP/TAZ-TEAD transcriptional activity. SWTX-143 demonstrates efficacy in Hippo-mutant tumor cells and induces substantial regression in human mesothelioma xenografts and orthotopic mouse models (212). Parallelly, the irreversible TEAD inhibitor MYF-03–69 covalently occupies TEAD’s palmitoylation site, blocking YAP-TEAD complex formation and transcriptional activity while inhibiting malignant pleural mesothelioma growth (213). Additionally, TM2—a novel TEAD inhibitor class—effectively suppresses TEAD autopalmitoylation. TM2 monotherapy or MEK inhibitor combination exerts potent antiproliferative effects against YAP-dependent cancers (214).

Beyond 2-BP and TEAD inhibitors, diverse palmitoylation-targeting agents show therapeutic promise. Ras proteins (including N-Ras) require palmitoylation/depalmitoylation cycling for subcellular trafficking and oncogenicity. While broad lipase inhibitors like Palmostatin M (Palm M) lack specificity, ABD957 selectively covalently inhibits ABHD17 depalmitoylases. In human AML cells, ABD957 blocks N-Ras depalmitoylation with higher proteome selectivity than Palm M. ABD957 synergizes with MEK inhibitors to suppress N-Ras signaling and inhibit NRAS-mutant AML growth, suggesting ABHD17 inhibitors as targeted therapies for NRAS-driven cancers (215). The marine-derived compound bengamide C (BC) enhances antitumor immunity by reducing PD-L1 abundance and boosting T-cell cytotoxicity. BC inhibits DHHC3 activity, preventing PD-L1 palmitoylation and triggering its membrane-to-cytoplasm translocation and lysosomal degradation. BC combined with anti-CTLA4 significantly enhances antitumor T-cell responses (216). Artemisinin—a clinically approved antimalarial—demonstrates anticancer activity by covalently inhibiting ER palmitoyltransferase ZDHHC6. This reduces oncogenic NRas palmitoylation, disrupting its subcellular localization and attenuating proliferative signaling. Clinical trials are evaluating artemisinin’s anticancer potential (217, 218). In neuropathic cancer pain (NCP), chronic morphine use accumulates morphine-3-glucuronide, activating microglia via ERK1/2 signaling and apelin receptor (APLNR). NCP models show ZDHHC9 upregulation palmitoylates APLNR, preventing degradation. APLNR palmitoylation inhibitors reduce inflammatory cytokine release and morphine tolerance, suggesting combination therapy potential for cancer pain (219). Additional small molecules regulate palmitoylation machinery:BI-2531, etoposide, piperlongumine; RXC004, all-trans retinoic acid (RA); MEK-PI3K dual inhibitors. These agents offer promising cancer treatment strategies (220–223). Notably, natural compounds (lutein, 5-hydroxyflavone, 6-hydroxyflavone) exhibit higher binding affinity to DHHC20 than 2-BP, supporting their development as selective DHHC20 inhibitors (224).

Palmitoylation, a critical mechanism of post-translational modification, drives tumor drug resistance and immune evasion by regulating protein localization, stability, and signaling pathway activation. In EGFR-TKI-resistant lung cancer, drug-tolerant persister (DTP) cells rely on fatty acid oxidation (FAO) for survival. Here, dipeptidyl peptidase 4 (DPP4) enhances fatty acid uptake via carnitine palmitoyl transferase 1a (CPT1A) activation and sustains mitochondrial antioxidant function through the DPP4-MEK-Nrf2 axis, thereby promoting resistance (225). Similarly, palmitoylation at cysteine residues C104/C107 of Claudin 4 (CLDN4) in hepatocellular carcinoma (HCC) stabilizes its lipid raft anchoring by inhibiting clathrin-mediated endocytosis, activating the Notch pathway to drive lenvatinib resistance (226). Regarding immune checkpoints, palmitoylation-dependent membrane localization of PD-L1 requires DHHC3 activity. The natural small molecule benzosceptrin C (BC) inhibits DHHC3 enzymatic activity, triggering lysosomal degradation of PD-L1 and reversing T cell exhaustion (216). Synergistic mechanisms of combination strategies are recognized as promising approaches to overcome therapeutic resistance or enhance treatment efficacy. Studies demonstrate that combining palmitoylation inhibitors with targeted/chemotherapeutic agents can overcome tumor resistance. For instance, osimertinib combined with the DPP4 inhibitor sitagliptin significantly suppresses lung cancer DTP cell survival, reduces residual tumor burden, and decreases recurrence rates (225). Doxorubicin plus the palmitoylation inhibitor 2-bromopalmitate (2-BP) enhances osteosarcoma apoptosis via the ROS/CHOP pathway (227). Furthermore, combination strategies sensitize tumors to chemo/targeted therapies: the PPT1 inhibitor DC661 reverses sorafenib adaptive resistance in HCC cells by disrupting lysosomal acidification and autophagic flux, while inducing mitochondrial apoptosis (208); salvianolic acid B, targeting CLDN4, inhibits hepatic-to-biliary transition (HBT) and restores lenvatinib sensitivity in HCC (226). Combining palmitoylation inhibitors with immunotherapy also enhances immune responses. PPT1 inhibitors (e.g., hydroxychloroquine [HCQ], DC661) induce interferon-β secretion, promote M2-to-M1 macrophage polarization, reduce myeloid-derived suppressor cells (MDSCs), and synergize with anti-PD-1 antibodies to augment T cell-mediated killing in melanoma (228). Activation of the ADH1C/PPARα axis promotes fatty acid degradation, reduces TEAD1 palmitoylation levels, suppresses the Hippo pathway, and potentiates PD-1 blockade efficacy (229). Innovative combination strategies have been proposed. FDX1 upregulation combined with elesclomol-Cu induces cuproptosis in colorectal cancer by promoting DLAT lipoylation and oligomerization, while the p53 activator CP-31398 sensitizes this process via enhanced FDXR expression (230, 231). Drug repurposing combinations demonstrate efficacy: antimalarials (e.g., artesunate) and central nervous system (CNS) drugs (e.g., fluoxetine, fluphenazine) synergize with chemotherapeutic agents by downregulating PPT1 expression, exhibiting favorable safety profiles in non-tumoral cells (232). Clinical challenges remain. Firstly, target selectivity and off-target effects: functional redundancy within the palmitoyltransferase family (e.g., DHHCs) may limit efficacy when inhibiting single isoforms (e.g., DHHC3) (216). Developing highly selective inhibitors requires structural biology-based optimization while assessing impacts on normal lipid metabolism (e.g., CPT1A inhibition may impair FAO in cardiomyocytes) (225, 227). Secondly, drug delivery and biodistribution: poor tumor targeting of small-molecule inhibitors (e.g., 2-BP, DC661) restricts efficacy and increases systemic toxicity (208, 227). Nanoparticle delivery systems (e.g., HSA-POPC/chol/AMOs liposomes) improve tumor accumulation of inhibitors or nucleic acid therapeutics, as evidenced by synergistic suppression of pancreatic cancer with miR-21 silencing plus sunitinib (233). Thirdly, tumor heterogeneity and microenvironment adaptation: heterogeneity in ADH1C deficiency or spatial distribution of CLDN4^+^ cells in HCC contributes to variable treatment responses (226, 229). Spatial multi-omics and dual biomarkers (e.g., ADH1C/PPARα) should guide patient stratification (229). Notably, the double-edged effect of immune microenvironment modulation: PPT1 inhibition enhances T cell responses but lysosomal membrane permeabilization may release damage-associated molecular patterns (DAMPs), provoking localized inflammatory storms (208, 228). Optimizing dosing windows or combining immunomodulators (e.g., IL-6 antagonists) is crucial to balance efficacy and safety. Future directions include: developing dual-functional molecules such as bispecific antibodies simultaneously targeting palmitoyltransferases and immune checkpoints (e.g., anti-CKAP4/PD-L1) (77); implementing dynamic monitoring technologies like mass cytometry to quantify membrane palmitoylomes for guiding treatment timing (216); and optimizing intervention timing, as palmitoylation mediates early tolerance (e.g., DTP cells), necessitating combination therapy initiation during initial treatment (208, 225).

Beyond anticancer applications of palmitoylation inhibition, studies demonstrate that palmitoylation significantly enhances intracellular delivery of therapeutic molecules—including 5-carboxyfluorescein and doxorubicin—independent of their inherent membrane permeability. Fluorescence imaging and flow cytometry validate palmitoylation’s role in promoting cellular uptake of Tat-fluorescein conjugates and doxorubicin. Notably, palmitoylated Tat-doxorubicin conjugates exhibit significantly enhanced anticancer activity in cervical cancer cell lines (234).

## 7 Conclusion and future directions

This review comprehensively examines the multifaceted roles of palmitoylation in tumor biology, elucidating its fundamental regulatory mechanisms and functional consequences. As a critical post-translational modification, palmitoylation exerts profound influence on tumor cell behavior by governing core oncogenic processes including tumor initiation, progression, invasion, and metastasis; modulating key signaling pathways such as Hippo and Wnt/β-catenin through regulation of effector protein activity, subcellular localization, and stability; and directing the function of tumor-associated proteins that orchestrate proliferation, apoptosis, and migration. Furthermore, we demonstrate palmitoylation’s systemic impact on tumor microenvironment interactions and immune evasion mechanisms—notably its regulation of immune cell functionality and suppression of surveillance pathways. Critical questions remain regarding tissue-specific mechanistic variations across tumor types, crosstalk with other post-translational modifications, and interactions with stromal and immune microenvironment components. While emerging palmitoylation inhibitors show preclinical promise, their clinical translation requires rigorous validation of therapeutic efficacy and safety profiles. Moving forward, research should prioritize developing novel isoform-selective inhibitors and activators, exploring combinatorial approaches integrating palmitoylation-targeted agents with conventional therapies, and advancing biomarker-driven patient stratification for precision intervention. In summary, palmitoylation represents a master regulatory node in cancer pathogenesis whose continued mechanistic dissection and therapeutic exploitation hold significant potential for advancing oncology treatment paradigms and improving patient outcomes.

## Glossary

PATs: Palmitoyl acyltransferases
PPTs: Palmitoyl-protein thioesterases
ER: Endoplasmic reticulum
2-BP: 2-bromopalmitate
CRC: Colorectal cancer
CKAP4: Cytoskeleton-associated protein 4
APF: Antiproliferative factor
CSCs: Cancer stem cells
HBECs: Human bronchial epithelial cells
TNBC: Triple-negative breast cancer
SD: Sleep deprivation
PA-CoA: Palmitoyl-CoA
DLBCL: Diffuse large B-cell lymphoma
FASN: Fatty acid synthase
PCa: Prostate cancer
AR: Androgen receptor
LDHA: Lactate dehydrogenase A
ROS: Reactive oxygen species
EGFR: Epidermal growth factor receptor
OSCC: Oral squamous cell carcinoma
GSCs: Gglioblastoma stem cells
NEK7: NIMA-related kinase 7
EOC: Epithelial ovarian cancer
TCA: Tricarboxylic acid
MDH2: Malate dehydrogenase 2
PDAC: Pancreatic ductal adenocarcinoma
IGF-1: Insulin-like growth factor-1
IGF-1R: IGF-1 receptor
Flot-1: Flotillin-1
APT: Acyl protein thioesterase
EMT: Epithelial-mesenchymal transition.
CLL: Chronic lymphocytic leukemia
Nar: Naringenin
ER: Estrogen receptor
LP: Lipid pocket
HCC: Hepatocellular carcinoma
PA: Palmitic acid
CcRCC: Clear cell renal cell carcinoma
DRM: Detergent-resistant membrane
NAFLD: Non-alcoholic fatty liver disease
NSCLC: Non-small cell lung cancer
MCAM: Melanoma cell adhesion molecule
PORCN: Porcupine
MET: Mesenchymal-to-epithelial transition
Shh: Sonic Hedgehog
Hhat: Hedgehog acyltransferase
MSA: Mobility Shift Assay
AML: Acute myeloid leukemia
HPSCC: Hypopharyngeal squamous cell carcinoma
CDK5: Cyclin-dependent kinase 5
GSCs: Glioblastoma stem cells
NCP: Neuropathic cancer pain
PD-L1: Programmed Death-Ligand 1
IFN-I: Type I interferon
DOX: Doxorubicin
OxLDL: Oxidized low-density lipoprotein
AFT: Afatinib
Palm M: Palmostatin M
BC: Bengamide C
RA: Retinoic acid
PRMT1: Protein arginine methyltransferase 1
HGSOC: High-grade serous ovarian cancer
OXA: Oxaliplatin
